# Long-term male-specific chronic pain via telomere- and p53‑mediated spinal cord cellular senescence

**DOI:** 10.1172/JCI151817

**Published:** 2022-04-15

**Authors:** Arjun Muralidharan, Susana G. Sotocinal, Noosha Yousefpour, Nur Akkurt, Lucas V. Lima, Shannon Tansley, Marc Parisien, Chengyang Wang, Jean-Sebastien Austin, Boram Ham, Gabrielle M.G.S. Dutra, Philippe Rousseau, Sioui Maldonado-Bouchard, Teleri Clark, Sarah F. Rosen, Mariam R. Majeed, Olivia Silva, Rachel Nejade, Xinyu Li, Stephania Donayre Pimentel, Christopher S. Nielsen, G. Gregory Neely, Chantal Autexier, Luda Diatchenko, Alfredo Ribeiro-da-Silva, Jeffrey S. Mogil

**Affiliations:** 1 Department of Psychology, McGill University, Montreal, Quebec, Canada.; 2 Charles Perkins Centre, The University of Sydney, Camperdown, New South Wales, Australia.; 3 Department of Pharmacology,; 4 Faculty of Dentistry, and; 5 Bloomfield Centre for Research in Aging, McGill University, Montreal, Quebec, Canada.; 6 Department of Chronic Diseases and Ageing, Norwegian Institute of Public Health, Oslo, Norway.; 7 Department of Pain Management and Research, Oslo University Hospital, Oslo, Norway.; 8 Department of Anesthesia, McGill University, Montreal, Quebec, Canada.

**Keywords:** Neuroscience, Cellular senescence, Pain, Telomeres

## Abstract

Mice with experimental nerve damage can display long‑lasting neuropathic pain behavior. We show here that 4 months and later after nerve injury, male but not female mice displayed telomere length (TL) reduction and p53‑mediated cellular senescence in the spinal cord, resulting in maintenance of pain and associated with decreased lifespan. Nerve injury increased the number of p53‑positive spinal cord neurons, astrocytes, and microglia, but only in microglia was the increase male‑specific, matching a robust sex specificity of TL reduction in this cell type, which has been previously implicated in male‑specific pain processing. Pain hypersensitivity was reversed by repeated intrathecal administration of a p53‑specific senolytic peptide, only in male mice and only many months after injury. Analysis of UK Biobank data revealed sex-specific relevance of this pathway in humans, featuring male‑specific genetic association of the human p53 locus (*TP53*) with chronic pain and a male-specific effect of chronic pain on mortality. Our findings demonstrate the existence of a biological mechanism maintaining pain behavior, at least in males, occurring much later than the time span of virtually all extant preclinical studies.

## Graphical abstract

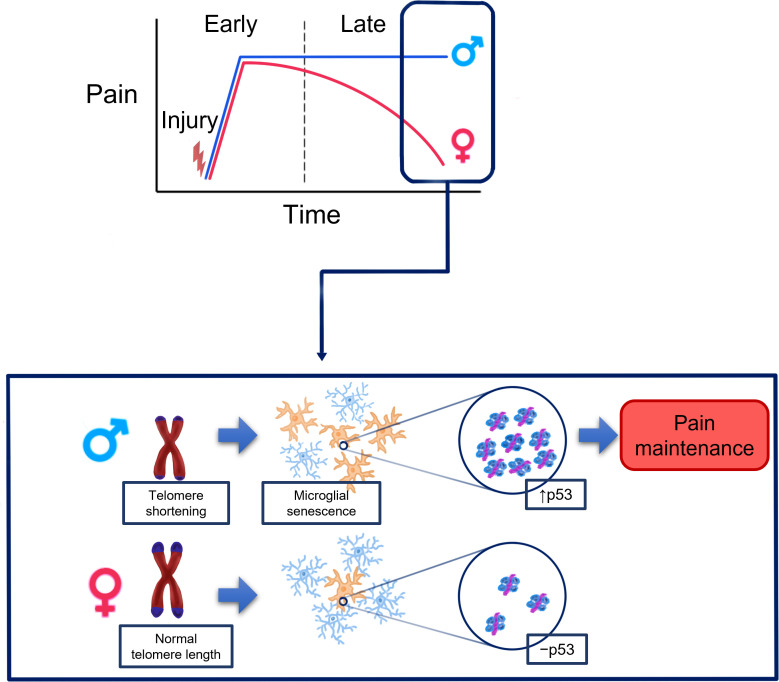

## Introduction

Chronic pain is the most prevalent human health problem, and among the most costly in terms of economic burden and loss of quality of life (1), but remains a relatively low priority for study in biomedicine, perhaps because it does not directly produce mortality. However, epidemiological investigations have revealed adverse effects of chronic pain on longevity, with the largest study thus far, based on the UK Biobank, demonstrating that chronic widespread pain is associated with excess all‑cause mortality relative risk of 1.59 even after adjustment for lifestyle factors (2). Strongly associated with mortality are the shortening and associated dysfunction of telomeres — complexes of tandem DNA repeats (TTAGGG) and proteins at the ends of eukaryotic chromosomes (3) — especially when death is caused by aging-related diseases (4). Chronic pain can be considered a disease of aging, and given the stressful nature of pain and the association of stress (5) and inflammation (6) with telomere shortening and dysfunction, it is perhaps not too surprising that reductions in leukocyte telomere length (TL) have been documented in patients with various chronic pain disorders in humans, including migraine (7), fibromyalgia (8), osteoarthritis (9), and endometriosis (10). Mechanisms underlying such a relationship remain entirely obscure.

Cellular senescence is a permanent state of cell cycle arrest induced by various cellular stressors, with the most common causal factor involving telomere shortening and dysfunction. The cellular growth arrest is established and maintained by the p53/p21 and p16^INK4a^ pathways, both finally converging on the retinoblastoma protein (pRb) (11). Cellular senescence is itself associated with a phenomenon called the senescence‑associated secretory phenotype, in which senescent cells release cytokines, chemokines, growth factors, and matrix remodeling factors, and attract immune cells, leading to a state of chronic inflammation (12). The role of telomere dysfunction–mediated senescence has been documented in various age-related human pathological conditions, such as atherosclerosis, Alzheimer’s disease, osteoarthritis, dementia, cardiovascular disorders, diabetes, and kidney disease (13). Although telomeres and cellular senescence have been implicated in the pathogenesis of diseases that feature pain (i.e., osteoarthritis and low back pain), a direct link between cellular senescence and chronic pain itself has never been made.

Both quantitative and qualitative sex differences in the genetic, neurochemical, and neuroimmune mediation of pain are known, and the introduction of sex-as-a-biological-variable policies have led to a recent explosion of such findings (14). We and others have demonstrated, in both mice and rats, that spinal cord microglia may play a male-specific role in pain over the course of several weeks after injury (15–18). It is known, however, that the pain hypersensitivity caused by some experimental nerve injuries lasts for many months. Mechanisms contributing to the maintenance of pain at time points so long after injury remain essentially unstudied.

Here we demonstrate a bidirectional relationship between telomeres and pain at time points long (≥4 months) after nerve injury, including causal evidence that genetically determined shorter telomeres can produce pain hypersensitivity, via p53‑dependent cellular senescence in the spinal cord. Furthermore, we observed an interaction between chronic pain and TL affecting mouse lifespan. Surprisingly, these relationships appear to be valid only in male mice, likely because of TL and senescence changes that occur in microglia. Evidence from the UK Biobank suggests male-specific relevance of these phenomena to chronic pain and mortality in humans.

## Results

### Peripheral nerve injury produces telomere shortening in mice in a manner that is dependent on duration, age, allodynia, and sex.

**Figure 1:**
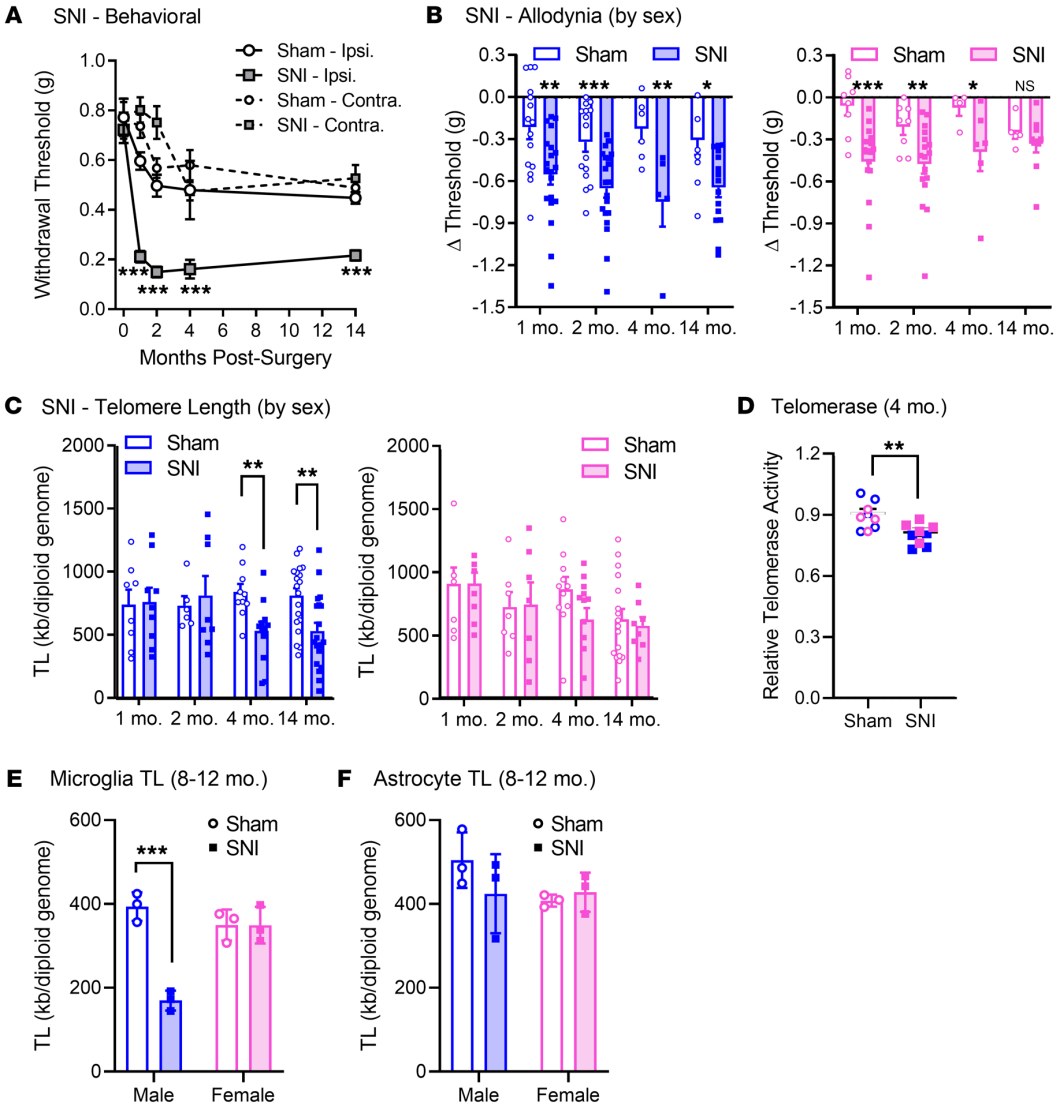
Relationship between TL and long-lasting allodynia produced by SNI. (**A**) SNI produces robust and long-lasting decreases in withdrawal thresholds of the ipsilateral (Ipsi.) hind paw compared with the contralateral (Contra.) hind paw and sham‑operated mice. Symbols represent mean ± SEM 50% withdrawal threshold (g); *n* = 24–38 mice/surgery/time point. Note that separate cohorts of mice were tested at each time point shown; baselines of all cohorts are averaged. (**B**) Allodynia data analyzed separately by sex (left/blue, males; right/pink, females). Bars represent mean ± SEM change (Δ) in withdrawal thresholds at each time point compared with baseline thresholds; data were transformed because baselines vary by sex. Compare with Supplemental Figure 1. (**C**) SNI leads to reductions in PBMC TL at 4 and 14 months after surgery in male mice (left) but not female mice (right). Bars represent mean ± SEM TL measured in kb/diploid genome; *n* = 16–36 mice/surgery/time point. (**D**) TL reduction at 4 months after SNI surgery is accompanied by lower telomerase enzyme activity in PBMCs. Symbols represent relative telomerase activity (see Methods). (**E** and **F**) Lumbar spinal cord FACS-sorted microglia (**E**) and astrocyte (**F**) TL data analyzed separately by sex. Bars as in **C**; *n* = 3 biological replicate pools/sex/surgery; each pool consisted of spinal cord tissues from *n* = 4 mice. *P < 0.05, **P < 0.01, ***P < 0.001 compared with other surgery group via *t* test.

Young adult (8-week-old) C57BL/6 mice of both sexes were given a spared (sural) nerve injury (SNI) to produce chronic neuropathic pain (see Methods). Robust, long-lasting decreases in withdrawal threshold of the ipsilateral hind paw (i.e., mechanical allodynia) were observed in mice receiving the injury compared with sham-operated mice (*P* < 0.001 compared with all other groups at all post‑surgery time points; Figure 1A), although some allodynia also gradually developed in sham-operated mice and on the contralateral hind paw of mice given SNI. Mechanical allodynia is the only reliably quantified long-lasting symptom in neuropathic assays (19). Further analysis of the behavioral data revealed a significant interaction with mouse sex at 14 months (*F*_1,33_ = 7.8, *P* = 0.009) after surgery. Although high levels of allodynia were maintained in male mice, SNI allodynia appeared to decrease over time in female mice, with a reduction apparent at 4 months and no significant difference from sham at 14 months (*t*_12_ = 0.8, *P* = 0.20; Figure 1B). This conclusion might be confounded by the fact that each time point represents a separate cohort of mice. Hence, we also confirmed the longer-lasting allodynia in male versus female mice in a separate experiment using a longitudinal design in which mice were tested repeatedly for over 5 months after SNI/sham (Supplemental Figure 1; supplemental material available online with this article; https://doi.org/10.1172/JCI151817DS1), and again in a recently published experiment in mice at 12 months after surgery (20).

Starting at 4 months after SNI, allodynia was accompanied by a significant reduction of peripheral blood mononuclear cell (PBMC) TL (4 months: *t*_44_ =3.3, *P* = 0.002; 14 months: *t*_62_ = 2.5, *P* = 0.02; Figure 1C). TL in these and subsequent experiments was quantified by an optimized PCR protocol; we confirmed the accuracy of this technique by comparing with a Southern blotting method using genomic DNA samples (Supplemental Figure 2). Paralleling the pain behavior data, the PBMC TL reductions after SNI appeared to be driven largely by male subjects, with statistically significant reductions in PBMC TL produced by SNI compared with sham surgery in male but not female mice at 4 months (male: *t*_20_ = 3.2, *P* = 0.005; female: *t*_22_ = 1.8, *P* = 0.09) and 14 months (male: *t*_37_ = 3.2, *P* = 0.003; female: *t*_23_ = 0.4, *P* = 0.69) after surgery.

Measurement of telomerase enzyme activity in the PBMCs of the 4-month cohort revealed lower levels in SNI- versus sham‑operated mice (*t*_16_ = 3.3, *P* = 0.004; Figure 1D). The reduction in relative telomerase activity in SNI-operated mice was also larger in males (sham vs. SNI: 0.92 vs. 0.78) than females (sham vs. SNI: 0.89 vs. 0.84), although not significantly so because of low sample size (surgery × sex: *F*_1,14_ = 2.5, *P* = 0.13).

Although TL correlates well among tissue types (21), microglia are the adult cell types in the central nervous system with the most significant mitotic potential, and thus are most susceptible to telomere shortening and potential cellular senescence (22). Microglia are also of particular interest in that we demonstrated a male-specific involvement of spinal cord microglia in the mediation of chronic pain using protocols very similar to those of the present study (15). In a separate cohort of male mice tested at 6 months after surgery, we confirmed that the allodynia was indeed reversible by intrathecal minocycline (Supplemental Figure 3). We thus checked to see whether TL was altered in microglia specifically. Using FACS, we isolated microglia from pooled lumbar spinal cords of sham- and SNI-operated mice 8–12 months after surgery. As shown in Figure 1E, SNI produced a robust reduction of TL in male but not female microglia (surgery × sex: *F*_1,8_ = 29.9, *P* = 0.0006). From the same tissue we also isolated astrocytes but observed no sex- or surgery-related effects on astrocyte TL (surgery × sex: *F*_1,8_ = 1.9, *P* = 0.20) (Figure 1F). We replicated the male‑specific microglial TL reduction in a separate experiment performed on spinal cord tissue collected from mice at 13 months after surgery (Supplemental Figure 4).

We also investigated the potential role of telomeres using a mouse model of inflammatory pain. Inflammatory injury via intraplantar injection of CFA also produced pain hypersensitivity compared with saline at 3 days after injection (drug × repeated measures: *F*_1,45_ = 9.3, *P* = 0.004), but this was not accompanied by TL reduction (*F*_1,25_ = 0.4, *P* = 0.48; Supplemental Figure 5). Testing of time points long after inflammatory injury is not possible because the hypersensitivity resolves within a few weeks.

Given the known relationship between TL and age (23), we hypothesized that the effect of pain on TL might be exaggerated in older mice. To assess this possibility, we conducted a similar experiment with young (2-month-old), middle‑aged (10-month-old), and older (20‑month‑old) male C57BL/6 mice. For this experiment we switched to another nerve injury model, the chronic constriction injury (CCI), both for purposes of generalizability and because the CCI features much more variable levels of mechanical allodynia. As shown in Supplemental Figure 6, PBMC TL was significantly reduced even 2 weeks after injury in older mice. In sum, these observations suggest a relationship between the pain produced by nerve injury and TL, although the causal direction cannot be inferred.

### Hypersensitivity to SNI allodynia of Terc-null mutant mice.

**Figure 2:**
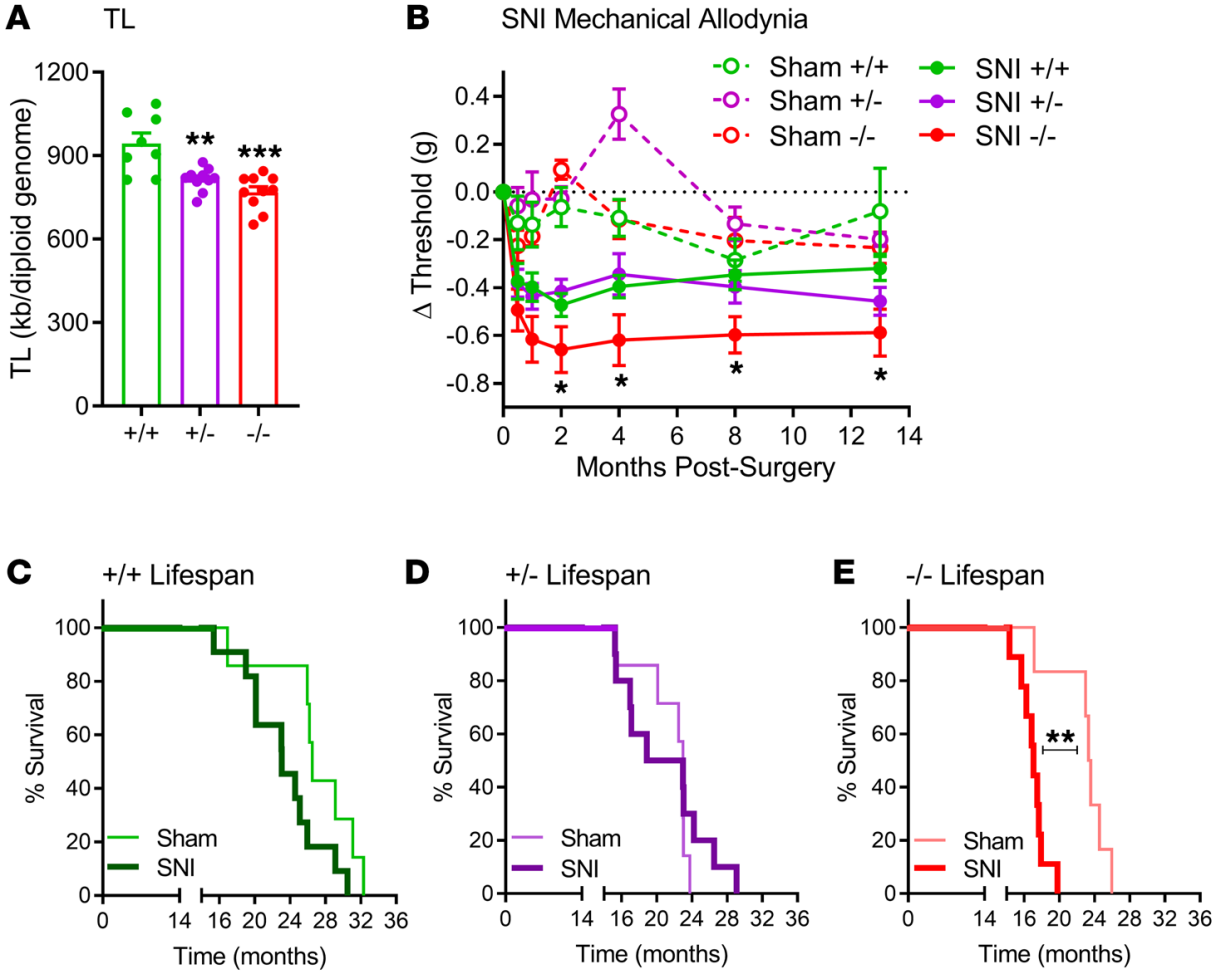
Pain and mortality phenotype of *Terc*-null mutant mice. (**A**) Confirmation of reduced PBMC TL in mice lacking 1 or 2 copies of the *Terc* gene. Bars represent mean ± SEM TL measured in kb/diploid genome; *n* = 8–10 mice per genotype. (**B**) Increased mechanical allodynia in the ipsilateral hind paw after SNI in *Terc^–/–^* mice. Symbols represent mean ± SEM change (Δ) in withdrawal thresholds (g) of the ipsilateral hind paw at each time point compared with baseline thresholds; *n* = 8–11 mice per genotype. (**C**–**E**) Kaplan-Meier survival curves of +/+ (**C**), +/– (**D**), and –/– (**E**) mice given sham or SNI surgeries at 2 months of age and left undisturbed (except for behavioral testing at time points shown in **B**) in their same-sex, same‑genotype home cages until death. *P < 0.05, **P < 0.01, ***P < 0.001 compared with corresponding sham group, +/+ genotype (within surgical condition), or as indicated, via 1-way ANOVA or log-rank test.

Telomeric DNA is maintained by telomerase, requiring telomerase RNA component (TERC), telomerase reverse transcriptase (TERT), and other binding proteins (24). Mice with a null mutation of the *Terc* gene display no detectable telomerase activity (24), but are viable over 4 generations, and adverse effects including decreased lifespan only occur in later‑generation mutants (25). We obtained first‑generation breeders from The Jackson Laboratory and mated them to produce wild-type (+/+), heterozygote (+/–), and second‑generation null mutant (–/–) mice. We first confirmed that *Terc^+/–^* and *Terc^–/–^* mice had lower PBMC TL than wild types, assessed at 12–18 weeks of age (*F*_2,25_ = 13.9, *P* < 0.001; Figure 2A). Mice of all 3 genotypes were then given SNI or sham surgery, and *Terc^–/–^* mice receiving SNI developed significantly increased long‑term mechanical allodynia in the ipsilateral hind paw (genotype × repeated measures: *F*_10,220_ = 2.2, *P* = 0.02; Figure 2B). The phenotype became statistically significant at 2 months after surgery. These data suggest, for the first time to our knowledge, a causal link between shortened telomeres and pain sensitivity. Although limited sample size precluded robust statistical testing, the increased SNI allodynia in *Terc^–/–^* mice was far more obvious in male subjects (Supplemental Figure 7).

### Terc genotype–dependent effect of chronic pain on mortality.

The last behavioral testing of the SNI- and sham-operated *Terc* mice was at 14–15 months of age. Mice were left undisturbed in their cages, and mortality was tracked. As shown in Figure 2, C–E, SNI caused early death in a *Terc* genotype–dependent fashion (sham vs. SNI log-rank test statistics; +/+: χ^2^ = 3.7, *P* = 0.052; +/–: χ^2^ = 0.4, *P* = 0.49; –/–: χ^2^ = 9.4, *P* = 0.002). Furthermore, the presence of SNI‑induced chronic pain exacerbated the effect of the *Terc-*null mutation on lifespan, since sham-operated mice did not display a significant effect of genotype (χ^2^ = 3.7, *P* = 0.15) whereas SNI-operated mice did (χ^2^ = 13.9, *P* = 0.001). Again the effect was primarily driven by male mice, as the effect of genotype on lifespan in SNI‑operated mice was significant in male (χ^2^ = 16.5, *P* = 0.0003) but not in female (χ^2^ = 2.8, *P* = 0.25) subjects (Supplemental Figure 8). The decrease in lifespan produced by SNI in male *Terc^–/–^* mice (6.2 months) exceeded the decrease in lifespan due to the *Terc*-null mutation itself (3.2 months).

### Peripheral nerve injury produces cellular senescence in the spinal cord of mice at time points long after injury.

**Figure 3:**
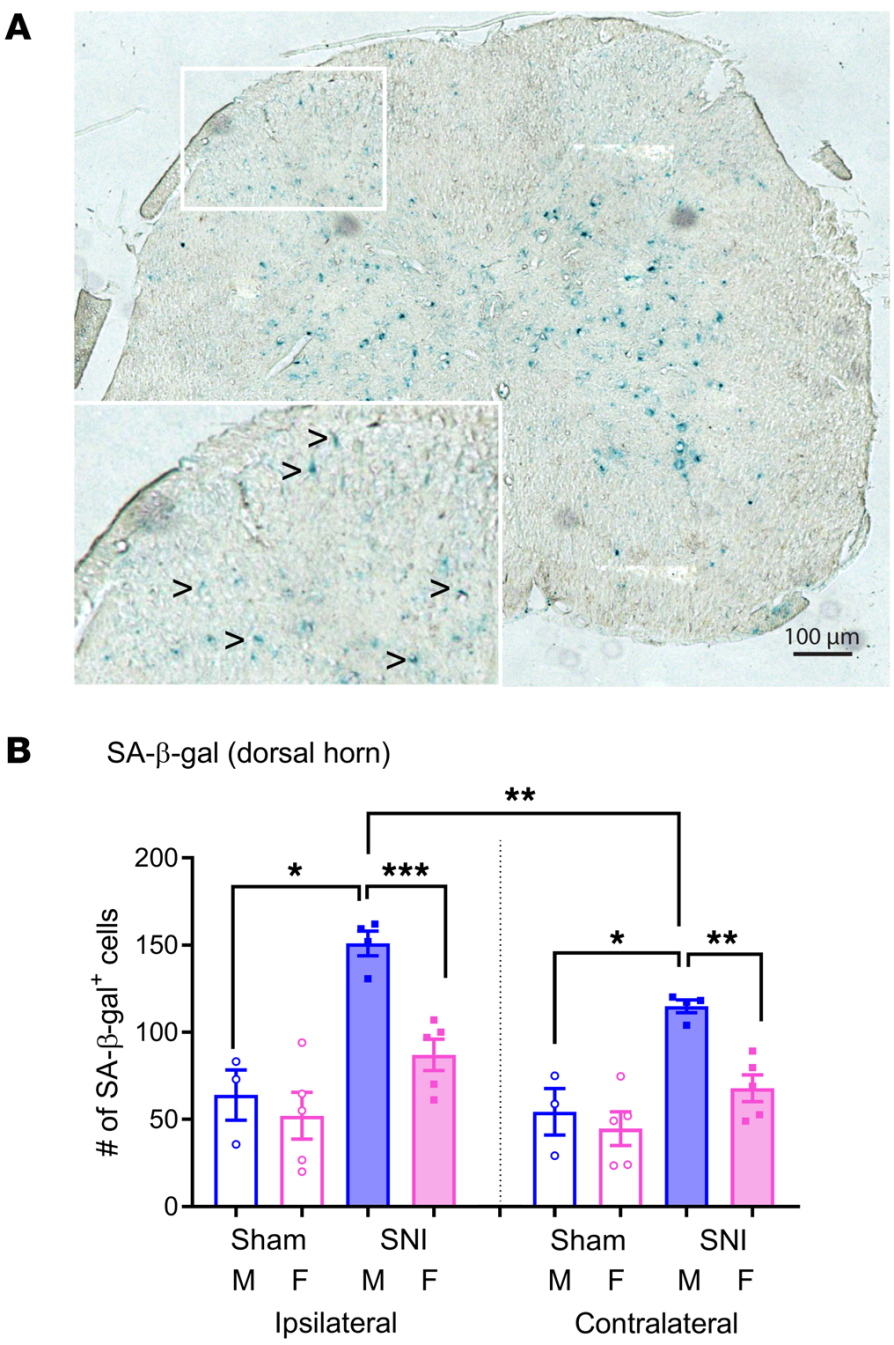
Peripheral nerve injury induces injury-, side-, and sex-dependent cellular senescence in the lumbar spinal cord of neuropathic mice long after injury. (**A**) Representative staining of SA-β-gal–positive cells in a lumbar spinal cord section from a mouse 14 months after SNI. The inset is an enlarged view of the lumbar dorsal horn; black arrowheads point to SA‑β‑gal–positive cells (turquoise). Scale bar: 100 μm. (**B**) Quantification of SA‑β‑gal–positive cells in the ipsilateral and contralateral dorsal horn of sham-operated and SNI mice of both sexes (M, male; F, female). Bars represent mean ± SEM number of positive cells; *n* = 3–5 mice/surgery/sex, with each symbol representing the average of 3–5 sections per mouse. *P < 0.05, **P < 0.01, ***P < 0.001 as indicated, by *t* test following 2-way ANOVA. Note that mice treated identically in which tissues were taken 2 months after SNI displayed zero detectable SA‑β‑gal–positive cells, and thus no representative section is shown.

Reduced TL can result in a persistent DNA damage response leading to cellular senescence (11, 26) — a state of cell cycle arrest/withdrawal, deregulated cellular metabolism, and macromolecular damage — and senescent cells in turn release a diverse set of cytokines, growth factors, proteases, and extracellular matrix components, together known as the senescence-associated secretory phenotype (SASP) or senescent-messaging secretome (12). Many of these SASP-related compounds are proinflammatory, and well known to produce or facilitate pain, especially when released in the spinal cord (27). We gave new cohorts of young male and female mice SNI or sham surgeries and harvested lumbar spinal cord tissues from these animals 12–14 months later, or 2 months later. Spinal cords were stained with senescence‑associated β‑galactosidase (SA‑β‑gal) to reveal putatively senescent cells (28). A representative spinal cord image is shown in Figure 3A; senescent cells were found throughout the spinal cord in the old cohort, but none at all in the young cohort (not shown). Quantification of SA‑β-gal–positive cells in the spinal cord dorsal horn revealed senescent cells in all groups (Figure 3B). A 2-between, 1-within repeated-measures ANOVA revealed a significant surgery × sex × side interaction (*F*_1,13_ = 5.1, *P* = 0.04), such that the highest number of positive cells were found in male SNI-operated mice. The same pattern was observed in superficial and deeper laminae of the dorsal horn (Supplemental Figure 9). The ventral horn also featured SA‑β-gal–positive cells, but in statistically equal numbers in all groups (*F*_1,13_ = 0.9, *P* = 0.36). Among a subset of mice tested both behaviorally and for SA‑β-gal staining, a highly significant correlation (*r* = –0.70, *P* = 0.002) was evinced between mechanical allodynia and the ipsilateral-contralateral difference in SA‑β‑gal–positive cells.

### Expression of senescence markers and SASP effectors in the spinal cord.

**Figure 4:**
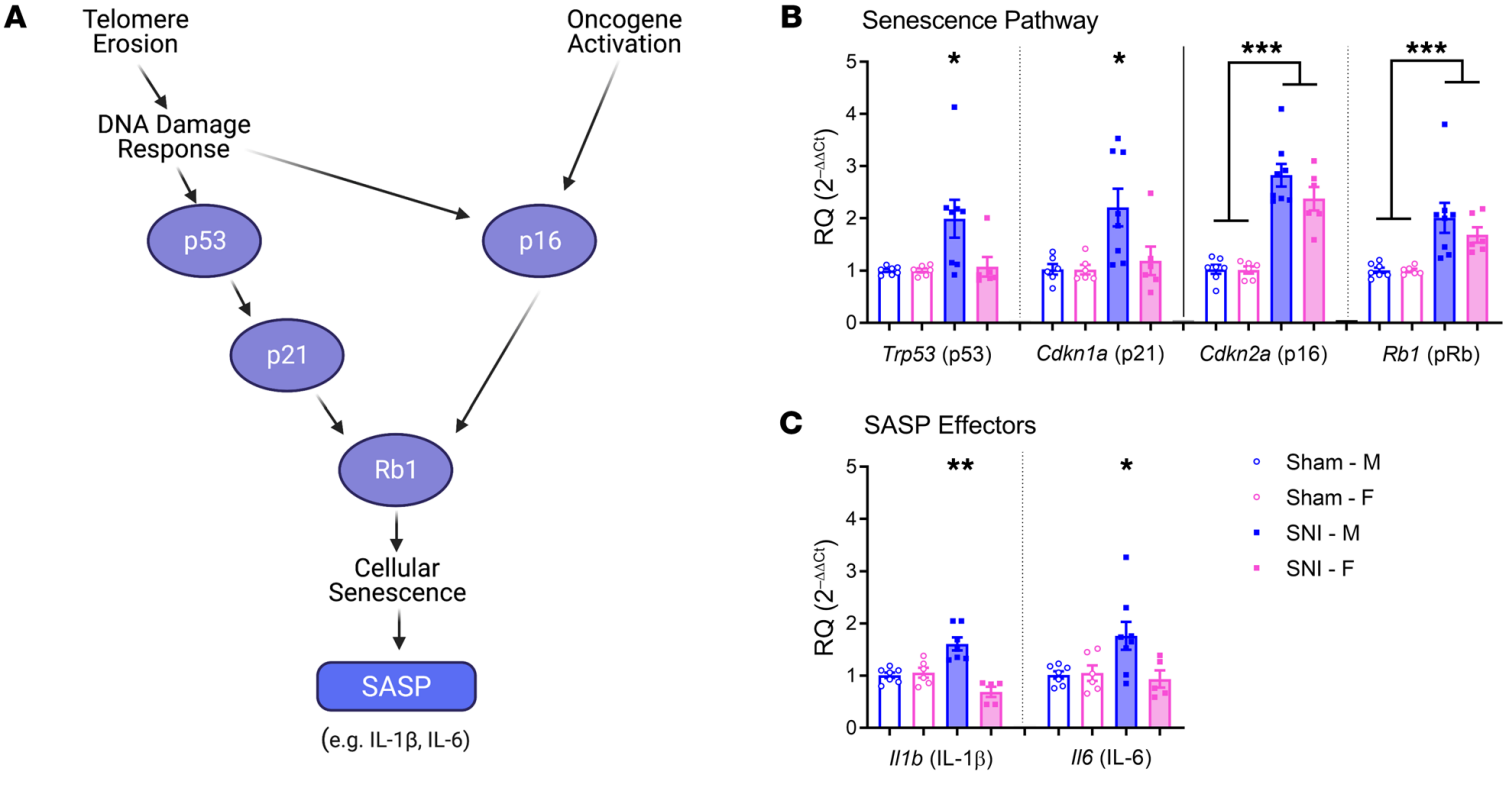
Expression of senescence pathway and SASP effector genes in the spinal cords of mice of both sexes 6 months after sham or SNI surgery. (**A**) Biological pathways leading to cellular senescence. (**B**) Expression of senescence pathway genes. (**C**) Expression of SASP effector genes. Bars in **B** and **C** represent mean ± SEM (*n* = 6–8 mice/surgery/sex; values shown are averages of 3 technical replicates) relative expression compared with the housekeeping gene, *Gapdh*, and normalized to the same-sex sham group. *P < 0.05, **P < 0.01, ***P < 0.001 compared with all other groups, or as indicated, via 2-way ANOVA.

The signal transduction systems leading to cellular senescence have been well studied; growth arrest is established and maintained by the p53/p21/Rb1 and p16^INK4a^/Rb1 pathways (ref. 29 and Figure 4A). The p53/p21 pathway is the more direct link from telomere shortening to senescence (30). Since SA-β-gal staining alone is not sufficient to unambiguously conclude that senescence is present, we obtained ipsilateral dorsal horn of the lumbar spinal cord tissue from mice of both sexes taken at 6 months after SNI or sham surgery, and performed real-time quantitative PCR (qPCR) on the *Trp53* (p53), *Cdkn1a* (p21), *Cdkn2a* (p16), and *Rb1* (pRb) genes, and the pain-producing cytokines *Il1b* (IL‑1β) and *Il6* (IL‑6). For p53 and its serial pathway partner, p21, SNI produced significantly higher gene expression in a male‑specific manner (both surgery × sex interactions: *F*_1,23_ = 4.6, *P* = 0.04; Figure 4B, left). For p16 and Rb1 genes, SNI produced increases equally in both sexes (surgery × sex interaction: *F*_1,23_ = 1.6, *P* = 0.22, and *F*_1,23_ = 0.7, *P* = 0.40, respectively; Figure 4B, right). Although pRb is also downstream of p53/p21, the increase observed in female mice with SNI was likely due to the p16 increase in this sex. Most importantly, we also observe a male-specific increase in gene expression of the major SASP effectors IL-1β and IL-6 (surgery × sex interaction: *F*_1,21_ = 24.8, *P* < 0.001, and *F*_1,22_ = 4.8, *P* = 0.04, respectively; Figure 4C).

### Cell type–specificity of SNI-induced p53 upregulation.

**Figure 5:**
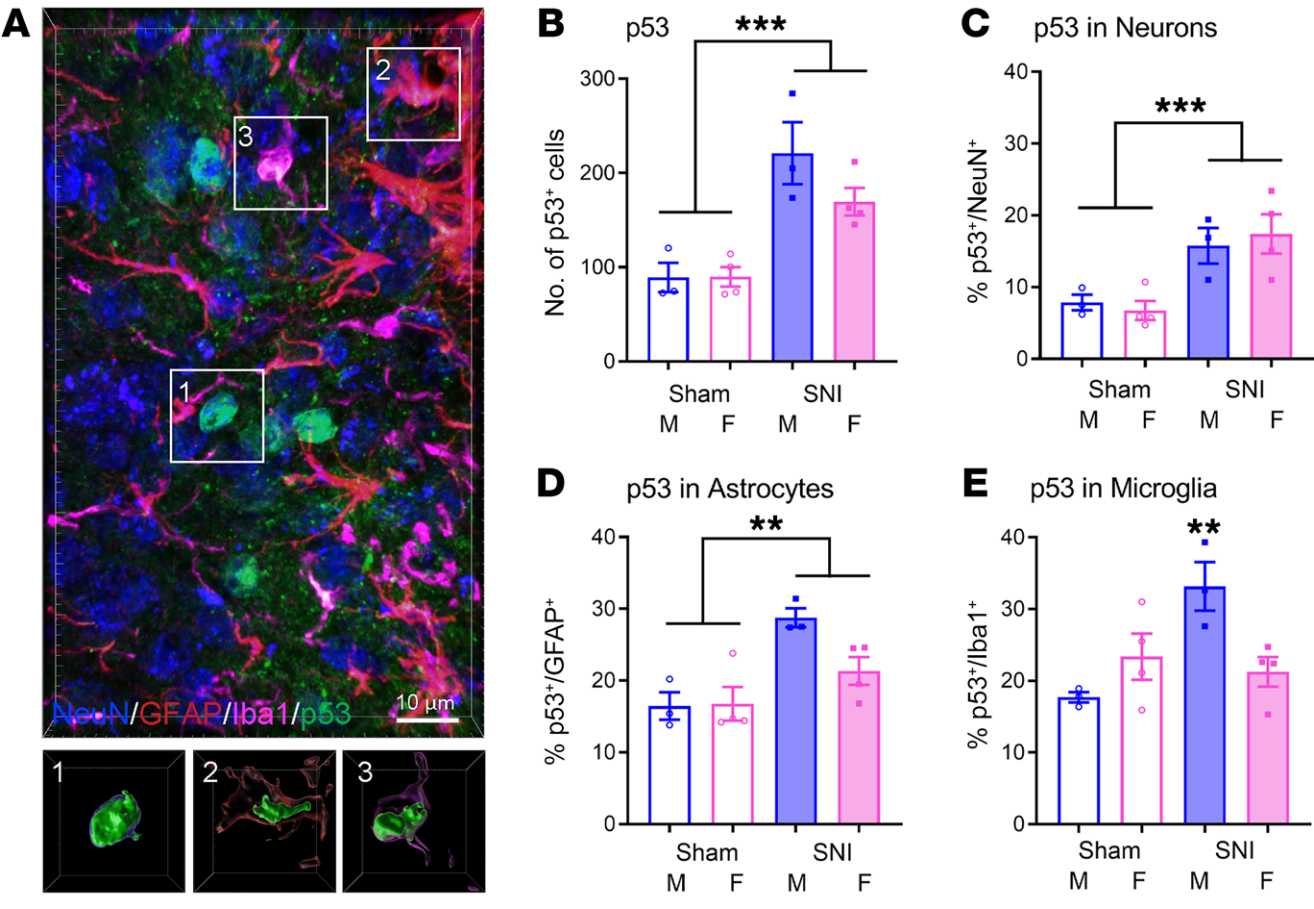
Sex-dependent upregulation of p53 by distinct spinal cord cell type 14 months after SNI. (**A**) Three-dimensional representative image of neurons (blue), astrocytes (red), microglia (magenta), and p53 (green) captured from the lumbar ipsilateral dorsal horn of an SNI-treated mouse. Insets in **A** show individual cells (1, 2, and 3) sampled from the image, and for each selected cell, a surface-rendered view is shown to demonstrate p53 expression in each cell type. Scale bar: 10 μm. (**B**) Quantification of p53‑positive cells by surgery (sham vs. SNI) and sex. Bars represent mean ± SEM number of cells containing p53 signal; *n* = 3–4 mice/surgery/sex, with each point representing an average of 3–7 scored sections per mouse. (**C**–**E**) Percentage of p53-positive cells also showing immunofluorescence for NeuN (**C**, neurons), GFAP (**D**, astrocytes), or Iba1 (**E**, microglia). Bars as in **B**. **P < 0.01, ***P < 0.001 compared with all other groups, or as indicated, via 2-way ANOVA.

Because it is extremely technically challenging to colabel SA-β-gal with antibodies, to determine the cell type–specificity of spinal cord senescence we used p53 expression as a proxy in double‑labeling immunohistochemistry experiments with NeuN or Nissl (for neurons), GFAP (for astrocytes), and Iba1 (for microglia). p53 staining was observed in lumbar dorsal horn spinal cord cells of all groups of mice 14 months after surgery. Shown in Figure 5A is a representative image of coexpression of p53 and cell type–specific markers in a mouse receiving SNI (see Supplemental Figure 10 for proof of antibody specificity). Quantification revealed a statistically significant main effect of surgery for p53-positive cells overall (*F*_1,10_ = 32.4, *P* < 0.001; Figure 5B). Quantification of p53 double labeling as a percentage of p53‑positive cells revealed significant main effects of surgery (but not sex, nor a significant surgery × sex interaction) for p53-NeuN (*F*_1,10_ = 19.0, *P* = 0.001) and p53-GFAP (*F*_1,10_ = 17.0, *P* = 0.002) (Figure 5, C and D). By contrast, for p53-Iba1 a significant surgery × sex interaction was evinced (*F*_1,10_ = 10.8, *P* = 0.008) (Figure 5E; also see Supplemental Figure 11B). Thus, only in microglia did SNI produce a clear male‑specific increase in p53 expression in accordance with the real-time qPCR data.

As p53 is also well known to mediate apoptosis (31), which might also affect chronic pain (32), we examined whether p53 colabeled with the apoptotic marker cleaved (activated) caspase-3 (CC3) in microglia. As shown in Supplemental Figure 11, although 15% of microglia show evidence of apoptosis via CC3, the percentage of microglia expressing CC3 was not increased by SNI in either sex. Since 25%–33% of microglia in male SNI mice were p53‑positive (Figure 5E and Supplemental Figure 11B), but only 13%–15% were CC3-positive (Supplemental Figure 11C), we can estimate that 16%–22% of microglia in the superficial ipsilateral dorsal horn were senescent at 14 months after nerve injury.

### p53-dependence of nerve injury–induced allodynia in male mice.

**Figure 6:**
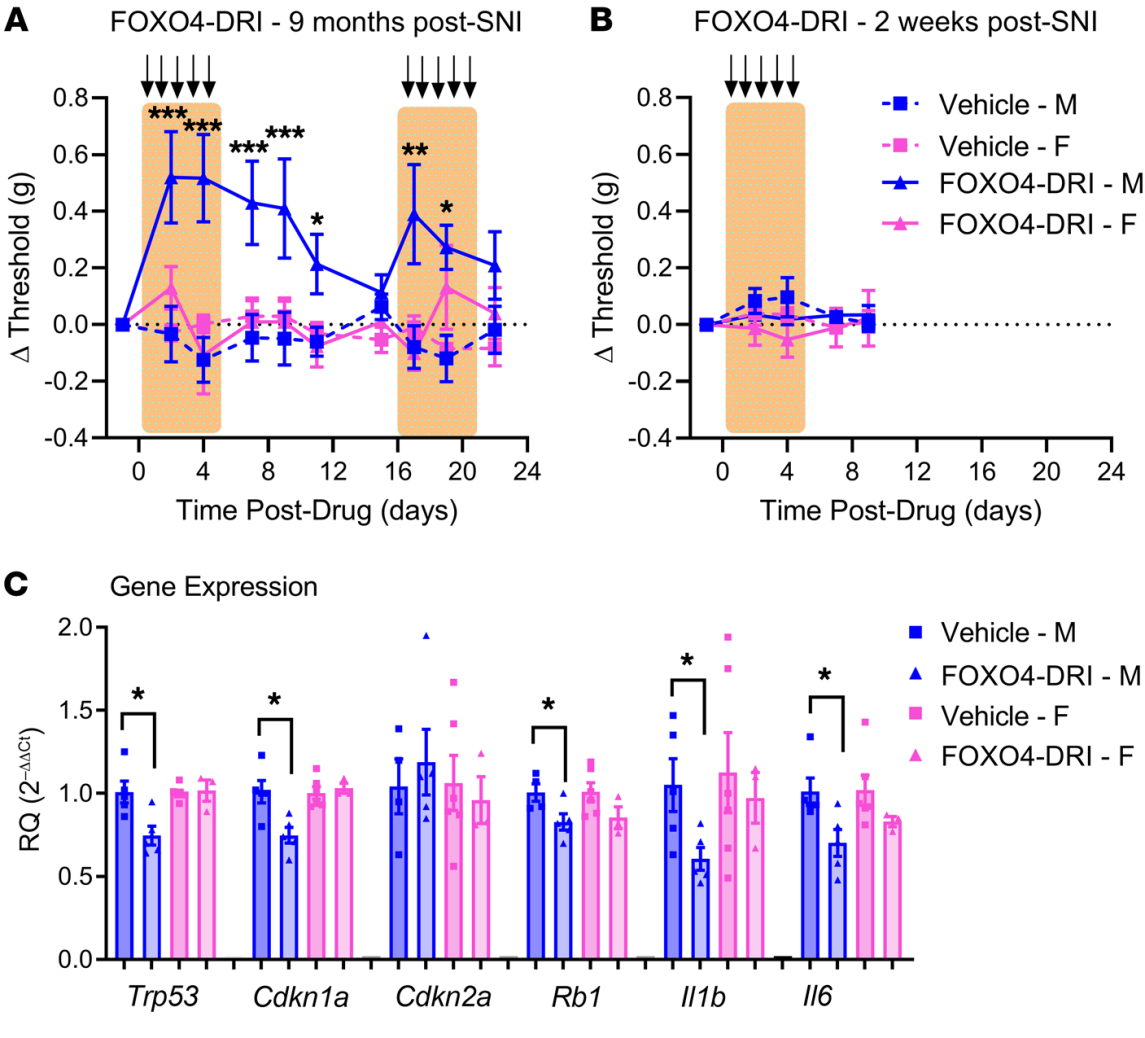
Targeted apoptosis of spinal cord p53-positive senescent cells using the peptide FOXO4-DRI reduces SNI allodynia in a sex- and time-dependent manner. (**A** and **B**) Mice of both sexes were tested for mechanical sensitivity and given SNI surgery. Nine months after SNI (**A**) or 2 weeks after SNI (**B**), they were tested again, and injected daily as shown by arrows with FOXO4-DRI (10 μg, intrathecally) or vehicle. Symbols represent mean ± SEM withdrawal threshold (g) of the ipsilateral hind paw (no changes were seen at any time point on the contralateral paw) compared with post-SNI testing immediately prior to drug administration (such that positive values represent reversal of mechanical allodynia); *n* = 5–6 mice/sex/drug/age. *P < 0.05, **P < 0.01, ***P < 0.001 compared with 0 (i.e., post-SNI, pre-drug baseline) via post hoc testing after 2-between, 1-within repeated-measures ANOVA. (**C**) Effect on senescence pathway and SASP effector gene expression of FOXO4‑DRI treatment in mice 9 months after SNI. Bars represent mean ± SEM (*n* = 3–6 mice/drug/sex; values shown are averages of 3 technical replicates) relative expression compared with the housekeeping gene, *Gapdh*, and normalized to the male-vehicle group. *P < 0.05 as indicated via *t* test following 2-way ANOVA.

The precise relationships between persistent DNA damage response signaling, p53, cellular senescence, and SASP are still a matter of debate, and we wished to provide causal evidence for the p53- and senescence‑dependence of pain hypersensitivity at time points long after injury. Thus, a specific test of the p53‑mediated senescence hypothesis was performed using the FOXO4 (forkhead box protein O4)–DRI (D‑retro inverso) peptide, which causes p53 nuclear exclusion in senescent cells, leading to their targeted apoptosis (33). Mice of both sexes were given SNI and left undisturbed for 9 months. As shown in Figure 6A, FOXO4‑DRI injected intrathecally (10 μg) once daily for 5 days reversed allodynia from 2 to 11 days after injection, and again upon reinjection, but only in male mice (drug × sex × repeated measures: *F*_9,153_ = 2.7, *P* = 0.006). Again, the FOXO4-DRI peptide had no effect in young mice (tested 2 weeks after SNI) of either sex (drug × sex × repeated measures: *F*_4,68_ = 0.7, *P* = 0.60; Figure 6B). To confirm the efficacy of the p53-specific targeting, we performed real-time qPCR on spinal cord tissue of mice 9 months after SNI and 22 days post-drug (i.e., immediately after cessation of behavioral testing shown in Figure 6A). We replicated the sex difference in SNI effects on p53, and the lack of a sex difference in p16 gene expression, at time points long after injury in vehicle-treated mice (see Supplemental Figure 12; compare with Figure 4B). As shown in Figure 6C, FOXO4-DRI significantly reduced p53, p21, and pRb gene expression in male but not female mice, as well as expression of the genes for downstream SASP effectors IL-1β and IL-6 (male: *t*_8_ = 2.5–3.2, *P* = 0.02–0.048; female: *t*_8_ = 0.3–1.7, *P* = 0.13–0.75) in the ipsilateral dorsal horn of the lumbar spinal cord. In contrast, chronic intrathecal administration of FOXO4-DRI did not affect gene expression of p16 in either sex (male: *t*_7_ = 0.5, *P* = 0.60; female: *t*_7_ = 0.4, *P* = 0.70). The male-specific reduction of p53 pathway genes is to be expected, as FOXO4-DRI only targets senescent p53-positive cells, which in turn would result in a decrease in Rb1 and SASP effectors. This experiment provides causal evidence that p53‑positive senescent cells in the spinal cord were maintaining pain hypersensitivity in male mice at late time points after injury.

### Human evidence from UK Biobank.

**Figure 7:**
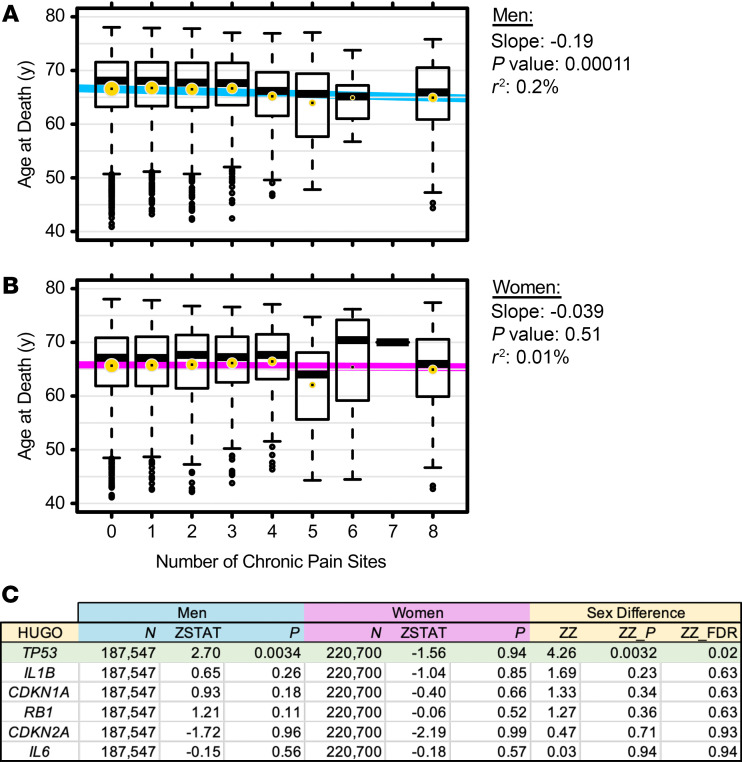
Human evidence from the UK Biobank. (**A** and **B**) Relationship between age at death and number of chronic pain sites, in men (**A**) and women (**B**). Slanted lines were obtained from linear regression. Box‑and-whiskers show age distributions for each number of pain sites; gold dots indicate means. (**C**) Gene-level summary statistics for sex-stratified GWAS on number of chronic pain sites. Genes (nomenclature according to the Human Genome Organization [HUGO]) considered were the human analogs of mouse senescence-related genes shown in Figure 4. *TP53* is the human analog of *Trp53*, encoding p53. *N*, sample size; ZSTAT, *z* statistic (MAGMA-assigned, gene-level summary test statistic; see Methods); *P*, 1-sided *P* value from test statistics; ZZ, difference between the men’s and women’s test statistics (men ZSTAT – women ZSTAT); ZZ_*P*, *P* value of ZZ, uncorrected; ZZ_FDR, false discovery rate–corrected *P* value of ZZ.

Although clear evidence exists that chronic pain can impact lifespan in humans (2), a sex‑stratified analysis of this relationship had not been performed. To do so, we used the large human cohort of the UK Biobank (34), in which a total of 14,421 deaths were recorded prior to June 2018. For these individuals, we compiled the number of pain sites from answers to self-reports of pain lasting for at least 3 months. In men, we found an inverse relationship between the number of concurrent chronic pain sites and age at death (Figure 7A). Each additional chronic pain site significantly decreased life expectancy by 0.2 years, or two-and-a-half months (*P* = 0.00011). This relationship was not observed in women (*P* = 0.51; Figure 7B).

We then searched for genetic evidence within the senescence-related biological processes shown to contribute to pain chronicity in male mice. We tested the effects of common SNPs within the 6 genes tested in Figures 4 and 6 on the quantitative phenotype of number of chronic pain sites in the UK Biobank cohort. We found that the *TP53* gene locus displayed a statistically significant genetic association with number of chronic pain sites in men (*P* = 0.0034), but not in women (*P* = 0.94) (Figure 7C).

## Discussion

In these experiments we observe a bidirectional interaction between TL and pain, in which long-lasting (≥4 months, or less in older mice) pain reduces TL, and genetically determined shorter telomeres are associated with increased pain sensitivity. The link between TL and pain appears to involve p53-mediated cellular senescence in the spinal cord, shown here by injury-, side-, and sex-dependent markers of senescence and SASP. The reversal of pain behavior by the modified peptide fragment FOXO4-DRI, which by competitively inhibiting the interaction between FOXO4 and p53 causes senescent cells to undergo apoptosis (33), further supports this notion. Intriguingly, the behavioral effects appear to be largely restricted to male mice, perhaps mediated by telomere shortening and senescence specifically in spinal cord microglia, despite the fact that senescence is widespread. Microglia from *Terc*-null mutant mice with short TL are known to display an enhanced proinflammatory response to lipopolysaccharide, likely the consequence of a compromised blood-brain barrier (35). The sex-specific and testosterone‑linked involvement of spinal cord microglia in pain hypersensitivity in rodents (15, 17) might thus explain the sex‑specificity of our current observations. An intriguing finding as well is the interactive effect of chronic pain and TL on mortality, an excellent example of gene‑by‑environment influences on telomere attrition and its sequelae (3). Evidence that similar processes may be occurring in our species includes the demonstration in over 400,000 individuals in the UK Biobank of a significant genetic association between the *TP53* gene (encoding p53) and chronic pain in men but not women, and a small but significant male-specific effect of widespread chronic pain on human lifespan.

That such effects can be demonstrated in the laboratory mouse is perhaps surprising given the long mean TL of this species and its short lifespan. We would note that robust changes in pain sensitivity were observed in only second-generation *Terc*-null mutants, at least 1 generation earlier than other abnormalities (25). Since stress‑induced senescence may be independent of telomerase activity and TL (36), it is possible that the TL changes seen here are only a marker of, and not causally responsible for, the pain phenotype.

To our knowledge, chronic pain–induced accumulation of senescent cells in the spinal cord has not been reported previously. Two recent reports have demonstrated that genetic and/or pharmacological elimination of senescent cells in the dorsal root ganglia or the articular cartilage can normalize cisplatin-induced mechanical allodynia (37) and weight bearing in an osteoarthritis model (38), respectively. We believe the failure to observe cellular senescence is likely due to the paucity of studies in which chronic pain has been followed out by experimenters past a few weeks after injury, and of pain studies using aged animals. Pain behavior after most inflammatory and neuropathic injuries in rodents generally resolves fully within 4–6 weeks. The exception is the SNI, which produces very long‑lasting mechanical allodynia (39), but even with this assay we are aware of no more than 18 published papers testing past the 3-month time point.

Other aspects remaining to be more fully explicated in future experiments include which feature of SNI decreases telomeres (i.e., nerve injury, pain, and/or stress), whether long‑lasting inflammatory pain can induce similar changes, the precise causes of death made more likely by SNI, the possible involvement of p16^INK4a^ cellular senescence pathways (in both sexes), the precise role of and requirement for p53 in senescence versus SASP (40), and which precise component(s) of the SASP are critical to maintaining pain hypersensitivity. This latter task is daunting, as so many individual SASP components have been individually shown to mediate pain. Although we surmise that the male-specific effects on pain may be explained by microglial involvement (15), why chronic pain would decrease telomeres only in males is unclear, although in a number of species, including mice and humans, telomeres shorten faster with age in males than females (41).

Women represent the clear majority of pain patients and have higher susceptibility to developing chronic pain than men (14), although whether the duration of chronic pain differs between the sexes in our species is not known. This study serves as another example that pain biology can differ robustly between the sexes and underscores the need to test both males and females routinely (42). Furthermore, our data suggest that clinically relevant pathophysiological mechanisms of “chronic” pain are being missed by the short testing timelines in current use in preclinical research. In humans, chronic pain is usually defined as that lasting 3 or 6 months after injury. The shorter lifespan of rodents leads to the assumption that chronic pain can be modeled in these species using post-injury time points of days to weeks. As the biochemical events underlying pain likely occur at approximately the same rate in all mammalian species, it is also reasonable to expect that acute‑to-chronic pain transitioning may actually occur at time points long after most extant preclinical pain studies have concluded. Thus, conducting preclinical pain research experiments at longer time points after injury may help improve translation of preclinical findings, and our data suggest the potential utility of senolytic drugs for the treatment of chronic pain, at least in men.

## Methods

### Mice

Male and female C57BL/6J mice were purchased from The Jackson Laboratory and used to breed mice of both sexes in-house. Heterozygous B6.Cg‑*Terc*^tm1Rdp^/J (stock 004132) breeders were obtained from The Jackson Laboratory) and were bred in‑house to generate second-generation *Terc*-null mutant mice. The DNA from tail samples was genotyped by the McGill University and Génome Québec Innovation Centre, per the protocol provided by The Jackson Laboratory, for the identification of wild-type (*Terc^+/+^*), heterozygous (*Terc^+/–^*), and homozygous (*Terc^–/–^*) mice. After weaning at 21–24 days of age, all mice were housed with their same-sex littermates in standard shoebox cages and maintained in a temperature-controlled (20°C ± 1°C) environment (12-hour light/12-hour dark cycle; lights on at 0700 hours), and received rodent chow (Envigo Teklad, 2920x) and tap water ad libitum.

### Algesiometry

Baseline bilateral hind-paw paw-withdrawal thresholds (PWTs) and the temporal development of mechanical allodynia in mice were assessed using von Frey fibers and the up‑down method of Dixon (43). Mice were placed individually in transparent Plexiglas cubicles (5 cm wide × 8.5 cm long × 6 cm high) placed on a perforated metal floor (with 5-mm-diameter holes placed 7 mm apart), and habituated for at least 2 hours before behavioral testing began. Nylon monofilaments (Stoelting Touch Test Sensory Evaluator Kit #2 to #9, corresponding to 0.015–1.3 g bending force; calibrated weekly using a microbalance) were firmly applied to the plantar surface of the hind paw (alternating the side of the body being tested) until they bowed for 5 seconds. Graphed values represent the mean of 2–3 threshold determinations per hind paw per time point. All experimenters were blinded to the genotype of the mouse and/or the surgery group; it was not possible to blind to sex. Mice were allocated to experimental conditions via a random-number generator.

In many experiments mice received a spared nerve injury (SNI), which was performed under isoflurane/oxygen anesthesia as described previously (44). We spared the sural nerve, and thus the lateral hind paw region innervated by the sural nerve was targeted with von Frey fibers. Sham-operated mice were anesthetized and incised to expose the sciatic nerve branches, but no nerves were transected.

In one experiment mice received a chronic constriction injury (CCI), which was performed under isoflurane/oxygen anesthesia as described previously (45). The mid‑plantar hind paw was targeted with von Frey fibers. Sham-operated mice were anesthetized and incised to expose the sciatic nerve, but no ligatures were introduced.

In one experiment, mice that had received an SNI injury 6 months prior were tested with von Frey fibers as described above, given an intrathecal injection (46) of minocycline hydrochloride (Sigma-Aldrich; 300 μg, 5 μL injection volume), and retested for mechanical withdrawal thresholds at 10, 20, 30, 60, and 120 minutes after injection.

### Isolation of mouse PBMCs

Blood samples (~0.7 mL) were obtained by intracardiac puncture and were mixed with 3 mL of PBS (pH 7.4) in a blood collection tube spray-coated with K_2_EDTA (BD Vacutainer). This mixture was then carefully layered over 4 mL of Histopaque 1083 (Sigma-Aldrich) solution in 15-mL conical tubes. The samples were then centrifuged at 18°C–20°C for 30 minutes (400 *g*; deceleration without brake), and the layer containing PBMCs was carefully pipetted out into 1-mL Eppendorf tubes. After subsequent washes with PBS (2 × 10 minutes), centrifugation (2 × 10 minutes at 400 *g*), and removal of supernatant solution, the PBMC pellet was processed further for DNA isolation using the DNeasy Blood and Tissue kit (Qiagen) per the manufacturer’s instructions. The quantity and quality of DNA were quantified using a NanoDrop spectrophotometer (Thermo Fisher Scientific).

### Isolation of mouse microglia and astrocytes

Fresh spinal cord samples (L4–L5 segment) were collected at 8–12 months after SNI or sham surgery from both male and female mice. One biological sample consisted of lumbar segments pooled from 4 mice. The microglia single-cell suspension protocol was adapted from ref. 47. Mice were deeply anesthetized and transcardially perfused with ice-cold HBSS. A 5-mm section of the spinal cord (lumbar region) was dissected and put into well plates containing 2 mg/mL collagenase type IV (Gibco) in 3 mL DMEM. Spinal cords were minced using scissors and treated with 1 μL of DNase I (Thermo Fisher Scientific) for 45 minutes under incubation at 37°C. At 20 minutes into the incubation, the tissue was Dounce-homogenized 20 times with a pestle. Cell suspensions were passed through a pre-wet (with DMEM) 70-μm cell strainer, transferred to a prechilled 50-mL tube, and centrifuged for 10 minutes at 22°C (400 *g*). Samples were washed by gentle decanting of the supernatant and addition of 15–20 mL 1× HBSS followed by centrifugation for 10 minutes at 22°C (400 *g*). The cell pellet was resuspended in 13 mL of 30% Percoll (Sigma-Aldrich) diluted in HBSS at room temperature. The 13‑mL cell suspension was transferred to a 15-mL tube and overlaid with 2 mL of HBSS. Cells were then centrifuged for 20 minutes at 22°C (400 *g*; zero brake). The debris and Percoll were carefully removed without disturbing the pellet of immune cells at the bottom of the tube. The cell pellet was washed with 15 mL of ice-cold HBSS and centrifuged for 7 minutes at 4°C (500 *g*). Samples were resuspended in 500 μL of ice‑cold FACS buffer (0.4% BSA [nonacetylated] in 1× PBS) with CD11b (PE), CD45 (APC‑Cy7), and ACSA2 (APC) and DAPI from BioLegend and Miltenyi Biotec at a 1:200 dilution for 1 hour on ice and protected from light. Samples were washed with ice-cold HBSS and centrifuged for 7 minutes at 4°C (500 *g*). Samples were sorted using either a FACSAria III cell sorter equipped with 405-nm, 488-nm, and 640-nm lasers and the appropriate filters (BD Biosciences), or a FACSAria Fusion equipped with 405-nm, 488-nm, 561-nm, and 633-nm lasers and the appropriate filters (BD Biosciences). In both cases, live, single CD11b^+^CD45^lo^ cells to label microglia, and ACSA2 to label astrocytes, were sorted using a 70-μm nozzle at 70 psi. Gates were determined using fluorescence minus one samples. FACS-sorted cells subsequently underwent DNA isolation using a Qiagen DNA Micro Kit (Qiagen, 56304). DNA was then cleaned up and concentrated using Zymogen DNA Clean and Concentrator (Zymogen Research, D4033).

### Telomere length analysis

#### Real-time qPCR assay.

The absolute telomere length (aTL) was assessed using real‑time qPCR technique, as previously described (48), with important modifications. Specifically, the concentrations of the telomere and 36B4 primers were reoptimized to improve the efficiency of the assay. Our optimization process also revealed that the use of Absolute QPCR SYBR Green Mix (Thermo Fisher Scientific, catalog AB1322) reduced interplate variability and improved the efficiency of this assay, in comparison with that of Power SYBR Green (Thermo Fisher Scientific), SYBR Green (Thermo Fisher Scientific), and HOTFIREPol EvaGreen qPCR SuperMix (Solis BioDyne) master mixes (data not shown).

The standard curves were generated from known quantities of synthesized HPLC-purified telomere (TTAGGG repeated 14 times; highest concentration standard of 400 pg), mouse 36B4 (5′‑ACTGGTCTAGGACCCGAGAAGACCTCCTTCTTCCAGGCTTTGGGCATCACCACGAAAATCTCCAGAGGCACCATTGA-3′; highest concentration standard of 170 pg), and human 36B4 (5′‑CAGCAAGTGGGAAGGTGTAATCCGTCTCCACAGACAAGGCCAGGACTCGTTTGTACCCGTTGATGATAGAATGGG-3′; highest concentration standard of 170 pg) oligonucleotides. The mouse and human telomere primers were 5′‑CGGTTTGTTTGGGTTTGGGTTTGGGTTTGGGTTTGGGTT-3′ (forward; 90 nM) and 5′‑GGCTTGCCTTACCCTTACCCTTACCCTTACCCTTACCCT-3′ (reverse; 90 nM). The mouse 36B4 primers were 5′-ACTGGTCTAGGACCCGAGAAG-3′ (forward; 300 nM) and 5′‑TCAATGGTGCCTCTGGAGATT-3′ (reverse; 300 nM). The human 36B4 primers were 5′‑CAGCAAGTGGGAAGGTGTAATCC-3′ (forward; 300 nM) and 5′‑CCCATTCTATCATCAACGGGTACAA-3′ (reverse; 300 nM). Plasmid DNA (*pBR322*) was added to each standard to maintain a constant total DNA per reaction tube. The concentration of standards, their molecular weights, and length in base pairs were then used to calculate the TL in kilobases and the number of copies of 36B4 in a diploid genome, as previously described (48).

The QuantStudio 3 and QuantStudio 7 real-time qPCR system was used with reaction conditions of 50°C for 2 minutes, followed by 95°C for 15 minutes, followed by 30 cycles of data collection at 94°C for 15 seconds, 57°C annealing for 10 seconds, and 72°C extension for 15 seconds along with melting curve from 60°C to 95°C. Reactions were carried out in 0.2-mL 96-well plates with both the telomere and 36B4 targets for samples run in triplicate. QuantStudio Design & Analysis software v1.4 was used for data analysis. The aTL in a diploid genome was calculated by division of the interpolated values of telomere kilobases per reaction by the diploid genome 36B4 copy number.

#### Telomere restriction fragment assay.

The telomere restriction fragment (TRF) assay was performed as previously described (49) with minor modifications. Briefly, 2.5–7.5 μg of human genomic DNA was digested with HinfI and RsaI restriction enzymes (New England Biolabs) and electrophoresed on a pulse field electrophoresis gel apparatus. HindIII-digested radiolabeled λ-DNA was included in each gel for TL calculation. The gel was hybridized with a (C_3_TA_2_)_3_ probe 5′-labeled with P32 by T4 kinase, overnight at 37°C. Radioactive signal was captured by phosphor screen, which was scanned on a Storm PhosphorImager and quantified using ImageQuant TL software. Mean TRF length was calculated by accounting for the higher signal intensity from larger TRFs, because of multiple hybridization of the telomere-specific hybridization probe, using the formula TRF = ∑(OD_i_)/∑(OD_i_/L_i_), where OD_i_ is the radioactive signal and L_i_ is the TRF length at position i.

### Telomeric repeat amplification protocol assay

The telomerase activity in mouse PBMCs was measured using the Telomerase TeloTAGGG PCR ELISA kit (Roche, catalog 11854666910), according to the manufacturer’s instructions. The telomeric repeat amplification protocol assay PCRs were performed in triplicate.

### SA-β-gal staining

Senescence-associated β-galactosidase (SA-β-gal) staining is the gold standard method for quantifying cellular senescence (50), and has recently been shown to sensitively measure changes in the spinal cord following injury in the mouse (28). Lumbar L4/5 spinal cord samples were isolated, postfixed in 4% paraformaldehyde for 2 hours, cryoprotected, and embedded in OCT medium. Frozen spinal cord sections (20 μm thick) were cut using a cryostat (Leica) and mounted onto Superfrost Plus slides. The Senescence β‑Galactosidase Staining Kit (Cell Signaling Technology, catalog 9860) was used to visualize the senescent cells, per the manufacturer’s instructions. Briefly, the lumbar spinal cord tissue sections were washed with 1× PBS (pH 7.4; 2 × 10 minutes) and were incubated with the SA‑β‑gal staining solution (pH 6.0) in the dark at 37°C (dry incubator; no CO_2_) for 24 hours. After the incubation period, the sections were washed with 1× PBS (3 × 10 minutes), coverslipped, and visualized using a Zeiss AxioImager M2 imaging microscope. Experimenters were blinded to the surgery group and sex of the mice during quantification.

### Real-time qPCR analysis of senescence and SASP markers

The ipsilateral dorsal horn of the lumbar L4/5 spinal cords was isolated and incubated in RNA*later* solution (Ambion). Subsequently, total RNA was isolated from the collected tissues using TRIzol Reagent (Ambion, catalog 15596026) and RNeasy Plus Universal Mini Kit (Qiagen, catalog 73404) and stored at −80°C until use. The cDNA was reverse-transcribed using the TaqMan Reverse Transcription Reagents kit (Thermo Fisher Scientific/Invitrogen, catalog N8080234). The mRNA levels of *Trp53* (Mm01731290_g1), *Cdkn1a* (Mm00432448_m1), *Cdkn2a* (Mm01257348_m1), *Rb1* (Mm00485586_m1), *Il6* (Mm00446190_m1), *Il1b* (Mm00434228_m1), and *Gapdh* (Mm99999915_g1) were analyzed using the TaqMan gene expression assay. Each reaction consisted of 15 μL of master mix (10 μL of TaqMan Fast Universal PCR Master Mix 2× [catalog 4352042], 1 μL of TaqMan assay, 1.5 μL of GAPDH Control Reagents [catalog 4308313], 2.5 μL of nuclease‑free water) and the reverse-transcribed cDNA (5 μL; 40 ng). Detection was performed on the QuantStudio 3 qPCR instrument. The samples were incubated at 50°C for 2 minutes, heated to 95°C for 20 seconds, and cycled at 95°C for 3 seconds and 60°C for 30 seconds for a total of 40 cycles. The analyses were performed using QuantStudio design and analysis software (v1.5.1) to determine the threshold cycle (Ct) values. The expression levels of target genes were normalized relative to mouse GAPDH expression, quantified using the ΔΔCt method, and averaged over 3 technical replicates per mouse. 

### Immunohistochemistry

Lumbar spinal cord pieces were cut on a cryostat (Leica) at −20°C, and 25‑μm‑thick free-floating sections were collected in PBS. Samples were subjected to antigen retrieval treatment with citrate buffer (1.92 g citric acid, 1 L ddH_2_O, pH 6.0) for 30 minutes at 90°C. After cooling for 20 minutes, sections were incubated in 50% ethanol (diluted with distilled water) for 20 minutes. Sections were then permeabilized with 0.2% Triton X in 0.01 M PBS (PBST) and blocked for 1 hour at room temperature in 10% normal donkey or goat serum. Sections were then incubated in a cocktail of primary antibodies in 5% blocking solution diluted in PBST for 12 hours at 4°C. Primary antibodies were rabbit anti-p53 (Abcam, catalog ab16665; 1:100), guinea pig anti-Iba1 (Synaptic Systems, catalog 234 004; 1:500), mouse anti-GFAP (Cell Signaling Technology, catalog 3670; 1:1000), guinea pig anti-NeuN (Millipore, catalog ABN90P; 1:1000), and mouse anti–cleaved caspase-3 (St. John’s Laboratory, catalog STJ97448; 1:100). Primary antibody labeling was detected using species-specific secondary antibodies conjugated to Alexa Fluor 488, Alexa Fluor 568, and Alexa Fluor 647 (Invitrogen; 1:800, incubated at room temperature for 2 hours). For the representative image (Figure 5), fluorescent Nissl (Invitrogen Neurotrace 435/455S, catalog N21479; 1:100) was used in the secondary antibody mix to label neurons. All sections were mounted on gelatin-subbed slides and coverslipped using Prolong Gold Antifade mounting medium (Invitrogen) and Zeiss coverslips.

### Microscopy

For p53 colocalization with different cell types, tissue sections from all experimental groups were imaged by Zeiss AxioImager M2 Imaging microscope with Zeiss ZenPro software v2.3 (Zeiss Canada). Acquisition settings were held constant for all images being compared by quantitative analysis. For representative images, *Z*-stacks were taken using a ×63 objective on a Zeiss LSM 880 confocal microscope. Three-dimensional and surface-rendered illustrations were prepared by Imaris software. Experimenters were blinded to the surgery group and sex of the mice during quantification.

### FOXO4-DRI

Male and female C57BL/6 mice had baseline PWTs measured at the age of 8 weeks. All mice were then given SNI surgery, and daily intrathecal injections of FOXO4 (forkhead box protein O4)–DRI (D‑retro inverso) peptide (Life Technologies) (10 μg in 5 μL saline) or vehicle (5 μL saline) were administered starting either 9 months or 2 weeks after SNI. PWTs were reassessed immediately before treatment and every 3 days starting after the first injection. In the 9-months-post-SNI group, a new series of injections was performed 11 days after the initial treatment period ended to investigate whether the analgesic effect could be recovered. Mice were euthanized at 22 days after the initial injection, and the ipsilateral dorsal horn of the lumbar spinal cord was isolated and processed for qPCR experiments.

### UK Biobank

The relationship between age at death and number of chronic pain sites — the best proxy available for chronic pain severity, which was not asked of participants — was explored using the large UK Biobank cohort (34, 51). For age at death, we used UK Biobank Field 40007 for data released circa June 2018 (14,421 recorded deaths). For sex we used UK Biobank Field 22001 (Genetic Sex): 8413 males, 5405 females, 603 undefined. The number of chronic pain sites assigned to a participant was the number of times that “yes” was answered to the questions “Have you had X pain for more than 3 months?”, indicating chronic pain in site X, in the following seven UK Biobank fields and sites: 3571, back pain; 4067, facial pain; 3799, headaches; 3414, hip pain; 3773, knee pain; 3404, neck/shoulder pain; 3741 stomach/abdominal pain. A participant could answer yes concurrently to any of these questions, for a value for the number of chronic pain sites between 0 and 7. Answering yes at field 2956, general pain, disabled all other pain sites; hence that participant was assigned a value of 8 for number of chronic pain sites. We performed linear regression of age at death versus number of chronic pain sites in a sex-stratified fashion.

A sex-stratified genetic association study of number of chronic pain sites was also conducted in the UK Biobank: 187,547 men, 220,700 women. Genetic analyses were conducted using BOLT (52), with the following covariates: 40 principal components to account for population stratification, age, age^2^, genotyping array, and dummy-coded recruitment sites. The number of chronic pain sites reported at the first assessment visit was used as a quantitative phenotype. Cryptic relatedness was accounted for by BOLT. Autosomal analyses were restricted to variants with a minor allele frequency greater than 0.1%, imputation INFO score greater than 0.8, and Hardy-Weinberg *P* greater than 10^–12^. Gene-level summary statistics were compiled using MAGMA (53), from SNP-level summary GWAS statistics. Each gene was assigned a test statistic, ZSTAT, from MAGMA’s *F* test. To uncover sex‑specific differences, we introduced a “distance” variable ZZ defined as ZSTAT_men_ – ZSTAT_women_. Genome-wide, ZZ was normally distributed; thus a *P* value for a specific ZZ value could be empirically assigned. The tested candidate genes, chosen because of the demonstrated relevance (or lack thereof, as a negative control) of their analogs in mice (see Figure 4), were: tumor protein p53 (*TP53*), cyclin-dependent kinase inhibitors 1A (*CDKN1A*; p21) and 2A (*CDKN2A*; p16), RB transcriptional corepressor 1 (*RB1*; pRb), IL-1β (*IL1B*), and IL-6 (*IL6*). Statistical significance was assessed at the FDR 10% level.

### Statistics

A criterion α level of 0.05 was adopted in all experiments. Data were analyzed by *t* test (2‑sided in all cases), ANOVA, or repeated-measures ANOVA, as appropriate, after determination of the normality (Shapiro-Wilk test) and homoscedasticity (Levene’s test) of the experimental data. Post hoc testing was performed using Tukey’s test or Dunnett’s test as appropriate. In 6 cases, data points were excluded as statistical outliers (standardized residuals >3); in no case does their inclusion alter conclusions.

Study approval

All in vivo experimental procedures in mice were approved by McGill University’s Animal Care and Use Committee and were performed in accordance with national guidelines. The UK Biobank study was conducted under UK Biobank application 20802, courteously initiated by Samar Khoury.

## Author contributions

AM and JSM conceived and designed experiments. AM, SGS, NY, NA, LVL, MP, CW, JSA, BH, ST, GMGSD, PR, SMB, TC, SFR, MRM, OS, RN, XL, and SDP performed experiments and/or analyzed data. CSN, GGN, CA, LD, and ARDS provided technical advice and edited the paper. AM and JSM wrote the paper.

## Supplementary Material

Supplemental data

## Version 1 04/15/2022

Electronic publication

## References

[2] Macfarlane GJ (2017). Persons with chronic widespread pain experience excess mortality: longitudinal results from UK Biobank and meta-analysis. Ann Rheum Dis.

[3] Blackburn EH (2015). Human telomere biology: a contributory and interactive factor in aging, disease risks, and protection. Science.

[4] Arbeev KG (2020). Association of leukocyte telomere length with mortality among adult participants in 3 longitudinal studies. JAMA Netw Open.

[5] Shalev I (2013). Stress and telomere biology: a lifespan perspective. Psychoneuroendocrinology.

[6] Jurk D (2014). Chronic inflammation induces telomere dysfunction and accelerates ageing in mice. Nat Commun.

[7] Ren H (2010). Shorter telomere length in peripheral blood cells associated with migraine in women. Headache.

[8] Hassett AL (2012). Pain is associated with short leukocyte telomere length in women with fibromyalgia. J Pain.

[9] Sibille KT (2017). Accelerated aging in adults with knee osteoarthritis pain: consideration for frequency, intensity, time, and total pain sites. Pain Rep.

[10] Sasamoto N (2020). Peripheral blood leukocyte telomere length and endometriosis. Reprod Sci.

[11] Gorgoulis V (2019). Cellular senescence: defining a path forward. Cell.

[12] Coppe JP (2010). The senescence-associated secretory phenotype: the dark side of tumor suppression. Annu Rev Pathol.

[13] Tuttle CSL (2021). Senescence in tissue samples of humans with age-related diseases: a systematic review. Ageing Res Rev.

[14] Mogil JS (2020). Qualitative sex differences in pain processing: emerging evidence of a biased literature. Nat Rev Neurosci.

[15] Sorge RE (2015). Different immune cells mediate mechanical pain hypersensitivity in male and female mice. Nat Neurosci.

[16] Sorge RE (2011). Spinal cord Toll-like receptor 4 mediates inflammatory and neuropathic hypersensitivity in male but not female mice. J Neurosci.

[17] Mapplebeck JCS (2018). Microglial P2X4R-evoked pain hypersensitivity is sexually dimorphic in rats. Pain.

[18] Taves S (2016). Spinal inhibition of p38 MAP kinase reduces inflammatory and neuropathic pain in male but not female mice: sex-dependent microglial signaling in the spinal cord. Brain Behav Immun.

[19] Mogil JS (2009). Animal models of pain: progress and challenges. Nat Rev Neurosci.

[20] Muralidharan A (2020). The influence of aging and duration of nerve injury on the antiallodynic efficacy of analgesics in laboratory mice. Pain Rep.

[21] Demanelis K (2020). Determinants of telomere length across human tissues. Science.

[22] Flanary BE, Streit WJ (2004). Progressive telomere shortening occurs in cultured rat microglia, but not astrocytes. Glia.

[23] Iwama H (1998). Telomeric length and telomerase activity vary with age in peripheral blood cells obtained from normal individuals. Hum Genet.

[24] Blasco MA (1997). Telomere shortening and tumor formation by mouse cells lacking telomerase RNA. Cell.

[25] Herrera E (1999). Disease states associated with telomerase deficiency appear earlier in mice with short telomeres. EMBO J.

[26] He S, Sharpless NE (2017). Senescence in health and disease. Cell.

[27] Ji RR (2014). Emerging targets in neuroinflammation-driven chronic pain. Nat Rev Drug Discov.

[28] Paramos-de-Carvalho D (2021). Targeting senescent cells improves functional recovery after spinal cord injury. Cell Rep.

[29] Campisi J (2013). Aging, cellular senescence, and cancer. Annu Rev Physiol.

[30] Herbig U (2004). Telomere shortening triggers senescence of human cells through a pathway involving ATM, p53, and p2^1CIP1^, but not p16^INK4a^. Mol Cell.

[31] Aubrey BJ (2018). How does p53 induce apoptosis and how does this relate to p53-mediated tumour suppression?. Cell Death Differ.

[32] Gao Y (2018). Bioinformatics analysis identifies p53 as a candidate prognostic biomarker for neuropathic pain. Front Genet.

[33] Baar MP (2017). Targeted apoptosis of senescent cells restores tissue homeostasis in response to chemotoxicity and aging. Cell.

[34] Sudlow C (2015). UK Biobank: an open access resource for identifying the causes of a wide range of complex diseases of middle and old age. PLoS Med.

[35] Raj DDA (2015). Enhanced microglial pro-inflammatory response to lipopolysaccharide correlates with brain infiltration and blood-brain barrier dysregulation in a mouse model of telomere shortening. Aging Cell.

[36] Hewitt G (2012). Telomeres are favoured targets of a persistent DNA damage response in ageing and stress-induced senescence. Nat Commun.

[37] Acklin S (2020). Depletion of senescent‑like neuronal cells alleviates cisplatin‑induced peripheral neuropathy in mice. Sci Rep.

[38] Jeon OH (2017). Local clearance of senescent cells attenuates the development of post-traumatic osteoarthritis and creates a pro-regenerative environment. Nat Med.

[39] Decosterd I, Woolf CJ (2000). Spared nerve injury: an animal model of persistent peripheral neuropathic pain. Pain.

[40] Johmura Y, Nakanishi H (2016). Multiple facets of p53 in senescence induction and maintenance. Cancer Sci.

[41] Barrett ELB, Richardson DS (2011). Sex differences in telomeres and lifespan. Aging Cell.

[42] Mogil JS (2016). Perspective: equality need not be painful. Nature.

[43] Chaplan SR (1994). Quantitative assessment of tactile allodynia evoked by unilateral ligation of the fifth and sixth lumbar nerves in the rat. J Neurosci Meth.

[44] Shields SD (2003). Spared nerve injury model of neuropathic pain in the mouse: a behavioral and anatomic analysis. J Pain.

[45] Bennett GJ (1988). A peripheral mononeuropathy in rat that produces disorders of pain sensation like those seen in man. Pain.

[46] Hylden JLK, Wilcox GL (1980). Intrathecal morphine in mice: a new technique. Eur J Pharmacol.

[47] Hammond TR (2019). Single-cell RNA sequencing of microglia throughout the mouse lifespan and in the injured brain reveals complex cell-state changes. Immunity.

[48] O’Callaghan NJ, Fenech M (2011). A quantitative PCR method for measuring absolute telomere length. Biol Proced Online.

[49] D’Souza Y (2013). Regulation of telomere length and homeostasis by telomerase enzyme processivity. J Cell Sci.

[50] Dimri GP (1995). A biomarker that identifies senescent human cells in culture and in aging skin in vivo. Proc Natl Acad Sci U S A.

[51] Verma V (2020). The dichotomous role of epiregulin in pain. Pain.

[52] Loh PR (2018). Mixed-model association for biobank-scale datasets. Nat Genet.

[53] de Leeuw CA (2015). MAGMA: generalized gene-set analysis of GWAS data. PLoS Comput Biol.

