# Supplementary material for: Long-term male-specific chronic pain via telomere- and p53‑mediated spinal cord cellular senescence
Source: J Clin Invest. 2022 Apr 15;132(8):e151817. doi: 10.1172/JCI151817 (PMC9012275; doi:10.1172/JCI151817)
Supplement: Supplemental data [file jci-132-151817-s175.pdf]

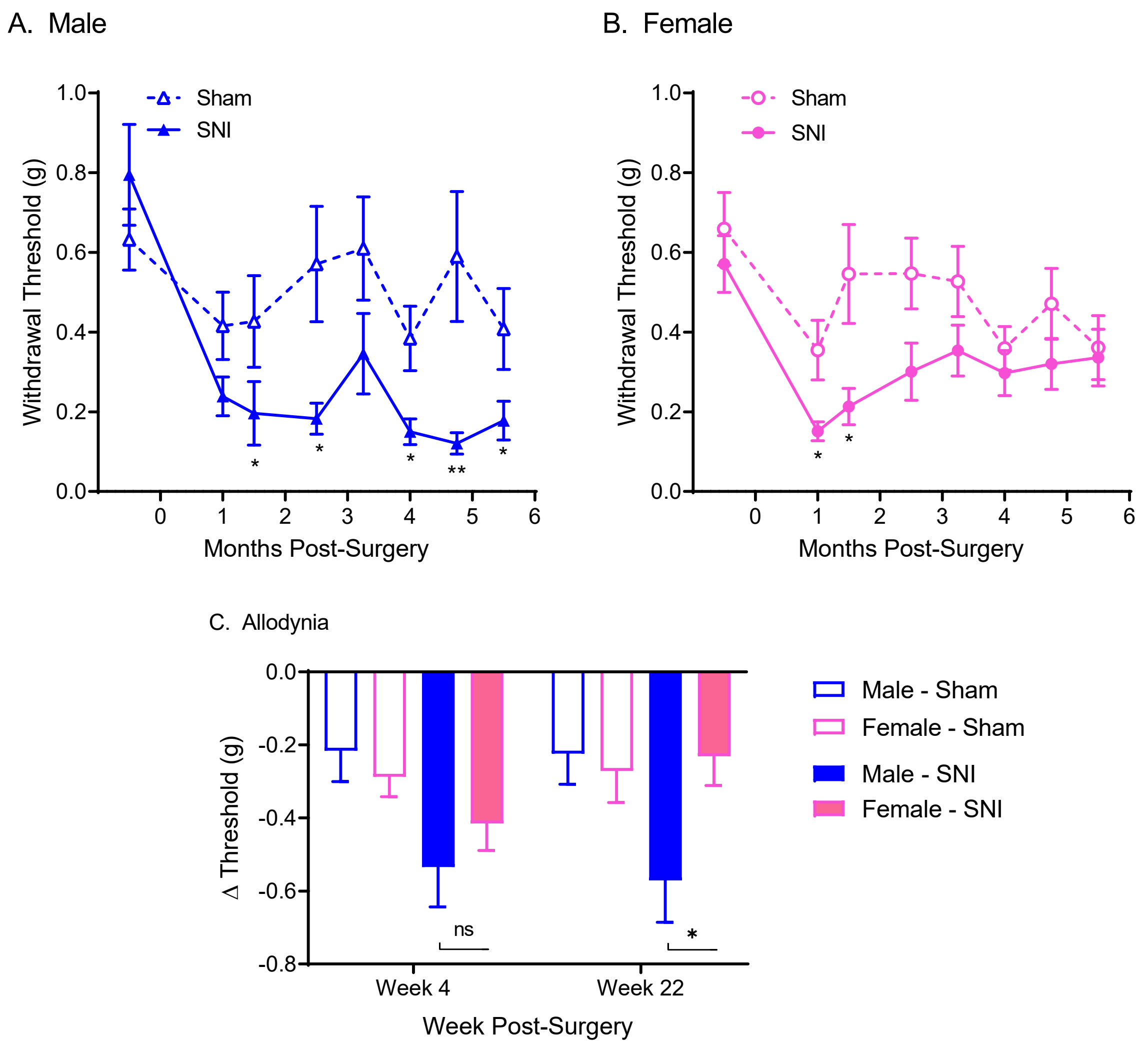

Supplementary Fig. 1. Confirmation in an independently performed experiment of the longer-lasting mechanical allodynia produced by SNI in male (A) versus female (B) mice. Compare to Fig. 1c. SNI produced significant allodynia in mice of both sexes (male repeated measures: $F_{7,91} = 13.8$, $p<0.001$; female repeated measures: $F_{7,161} = 5.2$, $p<0.001$). Sham surgery did not change withdrawal latencies in males (repeated measures: $F_{7,91} = 1.5$, $p=0.19$), but produced significant allodynia in females (repeated measures: $F_{7,154} = 3.8$, $p=0.001$). Symbols represent mean ± SEM hind paw withdrawal threshold (g); n=14–24 mice/sex/condition. **C**. Allodynia in all groups at the first and last post-surgical time points tested: 4 weeks and 22 weeks post-surgery. Symbols represent mean ± SEM change (Δ) in threshold (g) from baseline to the time point indicated. *$p<0.05$, **$p<0.01$ compared to sham at same time point or as indicated.

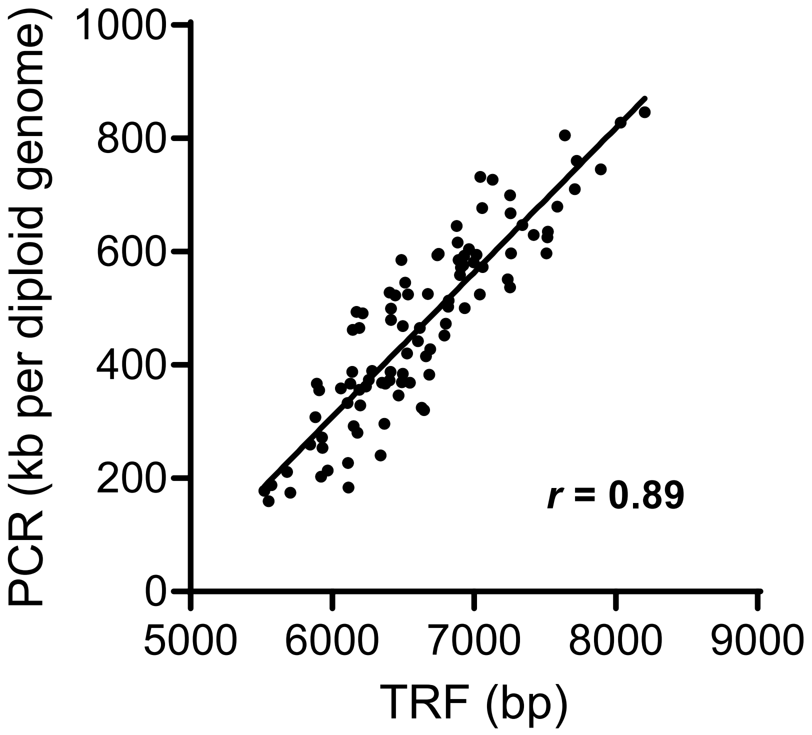

Supplementary Fig. 2. Correlation of human leuckocyte telomere lengths assessed using qPCR with Southern blot (TRF; see Methods). Data are from the blinded analysis of 132 genomic DNA samples.

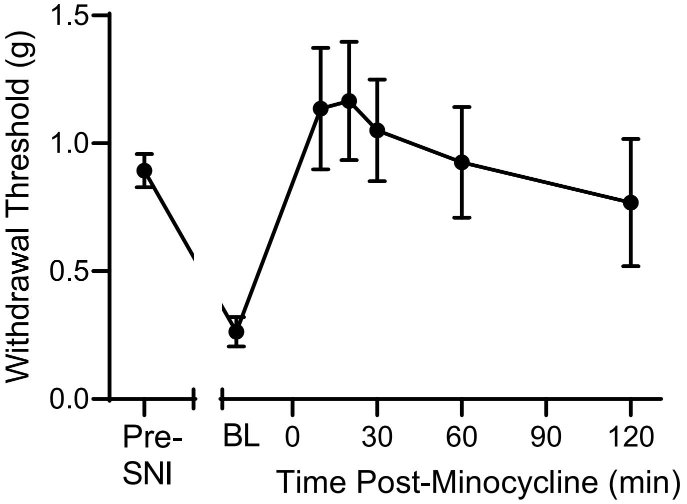

Supplementary Fig. 3. Sensitivity to minocycline of male mice at long after SNI. Symbols represent mean ± SEM withdrawal thresholds (g) before surgery (Pre-SNI), 6 months post-surgery (BL), and 10, 20, 30, 60, and 120 min after intrathecal injection of minocycline (300 µg in 5 µl). As can be seen, even at this time point post-SNI, mechanical allodynia is still robustly sensitive to minocycline reversal, suggestive of microglial-dependence.

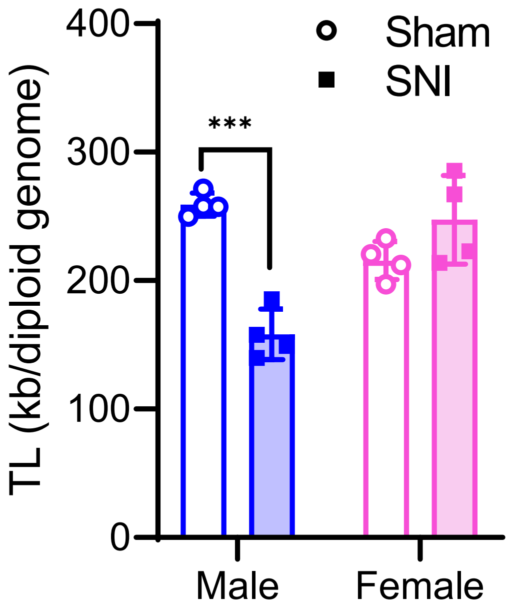

Supplementary Fig. 4. Replication of male-specific telomere length (TL) reduction caused by SNI. Microglia were isolated using fluorescence-activated cell sorting from pooled lumbar spinal cords of sham- and SNI-operated mice 13 months post-surgery. Bars represent mean ± SEM TL (kb/diploid genome). ***p<0.001 as indicated. Isolation of microglia was accomplished using a slightly different technique than described in the Methods section for the main experiment shown in Figure 1, E,F. For this experiment, spinal cord microglial cells were isolated using BD FACSAria Fusion™. Single-cell suspensions were prepared by digesting spinal cords in RPMI containing 1.6 mg/ml collagenase IV (Sigma), 200 µg/ml DNase I, and 2% Fetal bovine serum (FBS) for 30 min at 37 °C. Afterwards, samples were pressed through 70-µm nylon filter with a plunger, then RPMI was added to wash the filter. Homogenates were centrifuged at 400 *g* for 10 min. Pellets were resuspended in 5 ml of 30% isotonic Percoll, added on top of 4 ml of 70% isotonic Percoll in a 15-ml tube, then centrifuged for 20 min at 500x*g*. Immune cells were collected at the interface between the two layers of Percoll. The cells were washed with PBS and stained with anti-CD45 (eBioscience, Cat. #25-0451-82) and anti-CD11b (BD Biosciences, Cat. #562950) antibodies and viability dye. FACS-sorted cells subsequently underwent DNA isolation as described in Methods.

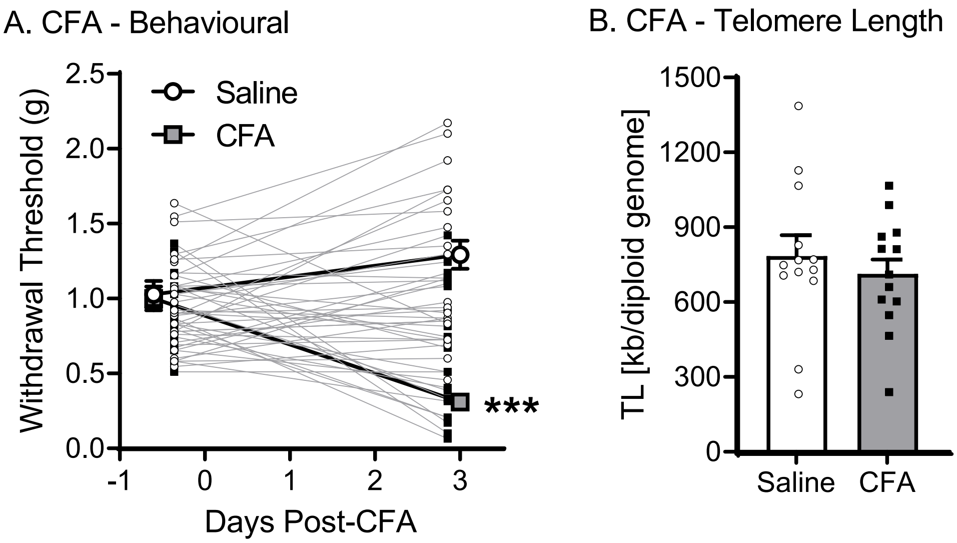

Supplementary Fig. 5. No change in telomere length (TL) produced by complete Freund's adjuvant (CFA) at 3 days post-injection. A) CFA produces mechanical hypersensitivity compared to saline. Symbols represent mean ± SEM withdrawal threshold in g using von Frey fibers; *n*=23–24 mice/condition. **B**) Bars represent mean ± SEM peripheral blood mononuclear cell TL in kb/diploid genome; *n*=13–14 mice/condition. ***$p<0.001$ compared to both CFA baseline and Day 3 saline.

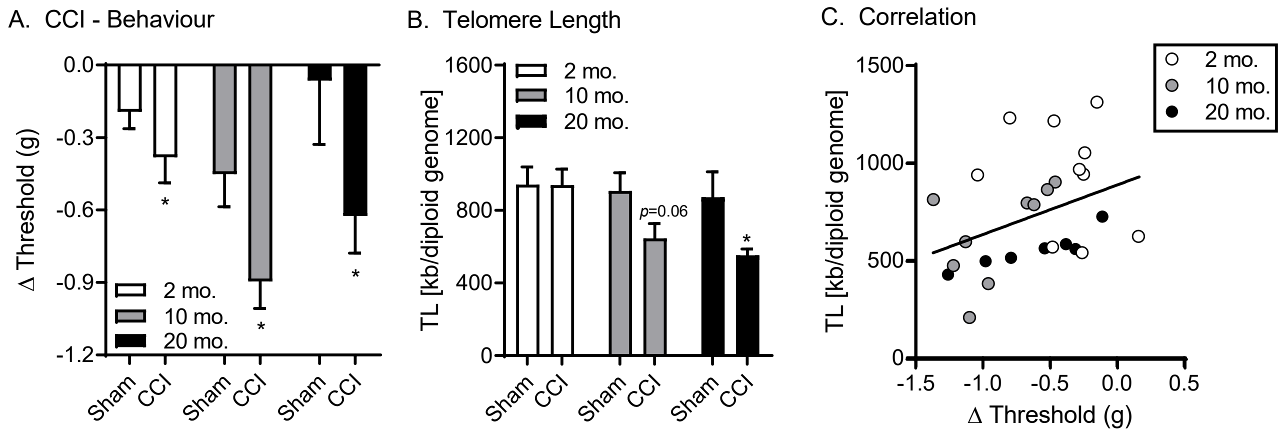

Supplementary Fig. 6. Influence of age on neuropathic pain behavior, telomere length (TL), and their correlation. Mice received a chronic constriction injury (CCI) or sham surgery at 2, 10 or 20 months (mo.) of age, and in every case was tested 14 days later. Bars in graph **A** represent mean ± SEM change in ipsilateral hind paw withdrawal threshold (g) compared to baseline; *n*=6–9 mice/age/surgery. *t*-test results were: 2 mo: t_{16} = 2.2, p=0.04; 10 mo: t_{13} = 2.5, p=0.03; 20 mo: t_{10} = 2.4, p=0.04. Bars in graph **B** represent mean ± SEM TL of peripheral blood mononuclear cells harvested immediately after behavioral testing. *t*-test results were: 2 mo: t_{16} = 0.1, p=0.92; 10 mo: t_{13} = 2.0, p=0.06; 20 mo: t_{10} = 2.6, p=0.03. The correlation between these measures in CCI-operated mice (r = 0.42, p=0.004) is shown in graph **C**. Note that the correlation is driven entirely by the 10- and 20-mo. old mice. Sham-operated mice (not shown) showed no such correlation (r = -0.28, p=0.25). *$p<0.05$ compared to corresponding Sham by Student's *t*-test.

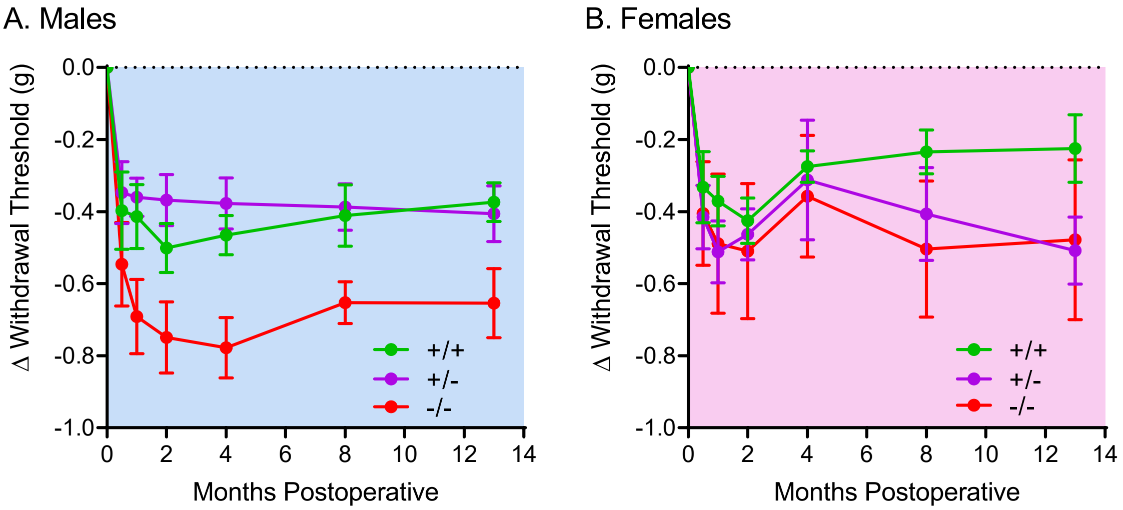

Supplementary Fig. 7. The increased mechanical allodynia caused by SNI (sham surgery groups not shown) in *Terc* null mutant (-/-) mice compared to wildtype (+/+) and heterozygotes (+/-) is more robust in male (a) compared to female (b) mice. Symbols represent mean ± SEM change (Δ) in hind paw withdrawal threshold (g) from baseline; n=5–7 mice/sex/genotype. Due to limited sample size, at no time point was a statistically significant genotype x sex interaction obtained using ANOVA (all p>0.05).

A. Males Only

B. Females Only

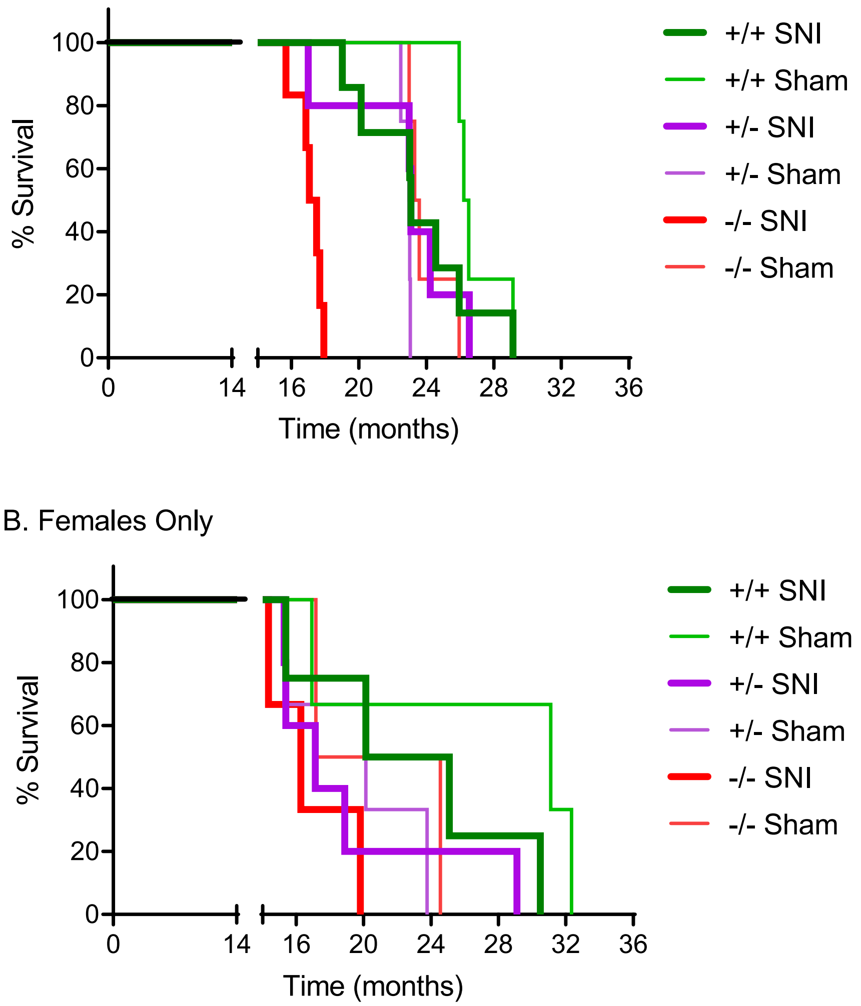

Supplementary Fig. 8. Mortality of SNI- and sham-operated *Terc* null mutant (-/-) compared to heterozygote (+/-) and wildtype (+/+) mice of each sex. The decreased life-span of SNI-operated -/- mice is much more obvious in male mice (**A**) than female mice (**B**); see main text for relevant statistics. Sample sizes range from *n*=6–11 mice/sex/genotype/surgical condition.

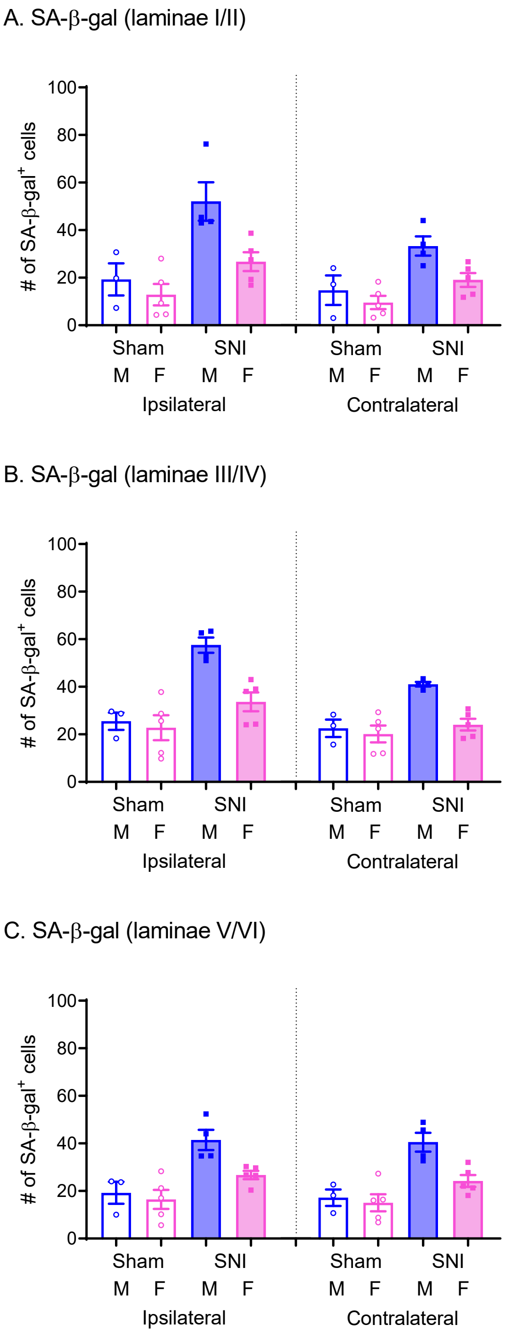

Supplementary Fig. 9. Senescent-associated β-galactoside-positive (SA- β -gal+) cells in various laminae of the spinal cord dorsal horn. Shown are data from Fig. 3B broken down by laminae I/II (**A**), laminae III/IV (**B**), and laminae V/VI (**C**). In all graphs, bars represent mean ± SEM positive cells.

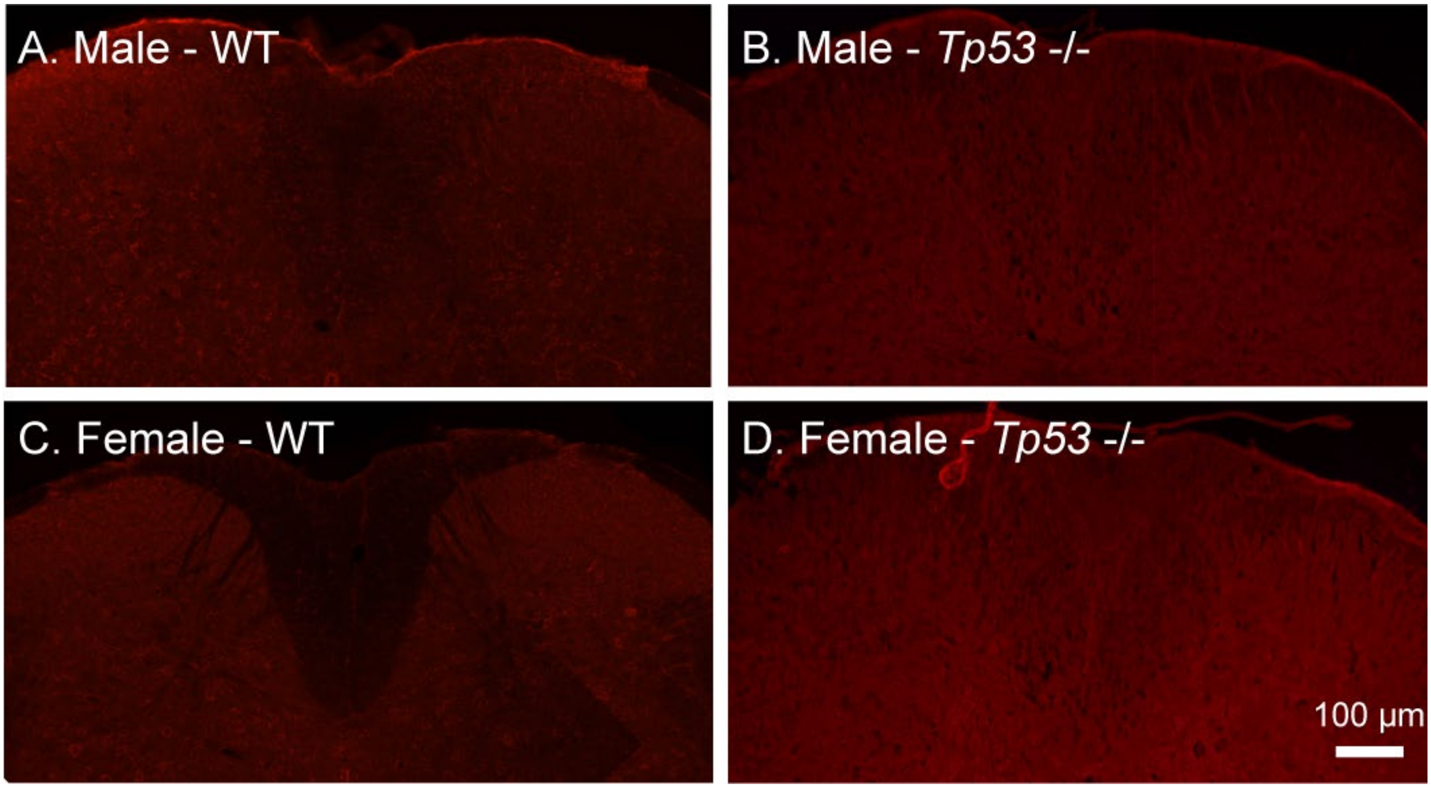

Supplementary Fig. 10. Absence of p53 immunoreactivity in neuropathic *Tp53* null mutant (-/-) mice validates antibody specificity. Low magnification images of dorsal spinal cord immunostained for p53 protein from wildtype (WT) (**A**,**C**) and *Tp53* (p53) -/- (**B**,**D**) male (**A**,**B**) and female (**C**,**D**) mice.

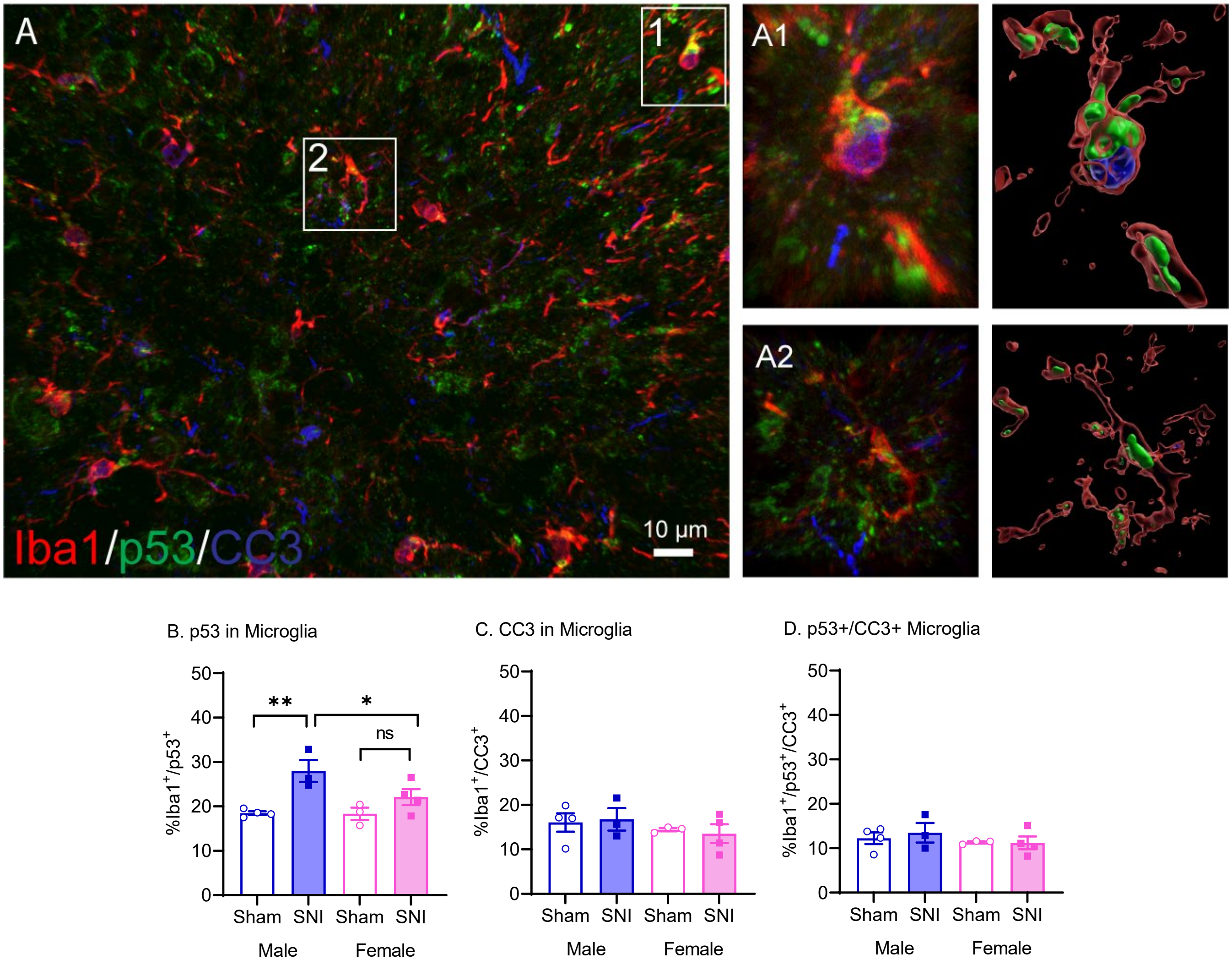

Supplementary Fig. 11. No evidence for surgery- or sex-dependence of apoptosis in microglia. **A**) Three-dimensional representative image of microglia (red), p53 (green), and CC3 (blue) captured from a dorsal horn section of a mouse 14 months after SNI shows co-expression of CC3 and p53 in a fraction of microglia. Examples of an apoptotic (a1: $p53^+/CC3^+$) and senescent (a2: $p53^+/CC3^-$) microglial cell are magnified and showed in the second column. For each enlarged image, a surface rendered view of the cell is shown (right column) to better demonstrate p53 and CC3 expression in microglia. **B**) An independent replication of the male-specific increase in p53+ microglia after SNI (compare to Fig. 4e). Two-way ANOVA revealed a significant surgery x sex interaction: $F_{1,10}$ = 5.1, p=0.04. **C**) No difference in percentages of microglia (Iba^+ cells) also expressing the apoptotic marker cleaved caspase 3 ($CC3^+$). No main effects nor interactions were noted (all p>0.05). **D**) No difference in percentages of microglia expressing both p53 and CC3. No main effects nor interactions were noted (all p>0.05). Bars represent mean ± SEM percentages of total microglia as indicated; n=3–4 mice/surgery/sex, with each point representing an average of 3–8 scored sections per mouse. *p<0.05, **p<0.01 as indicated; n.s., not significantly different. Since ≈27% of microglia in male-SNI mice are $p53^+$, but only ≈11% are $CC3^+$, we can estimate that 16% of microglia are senescent.

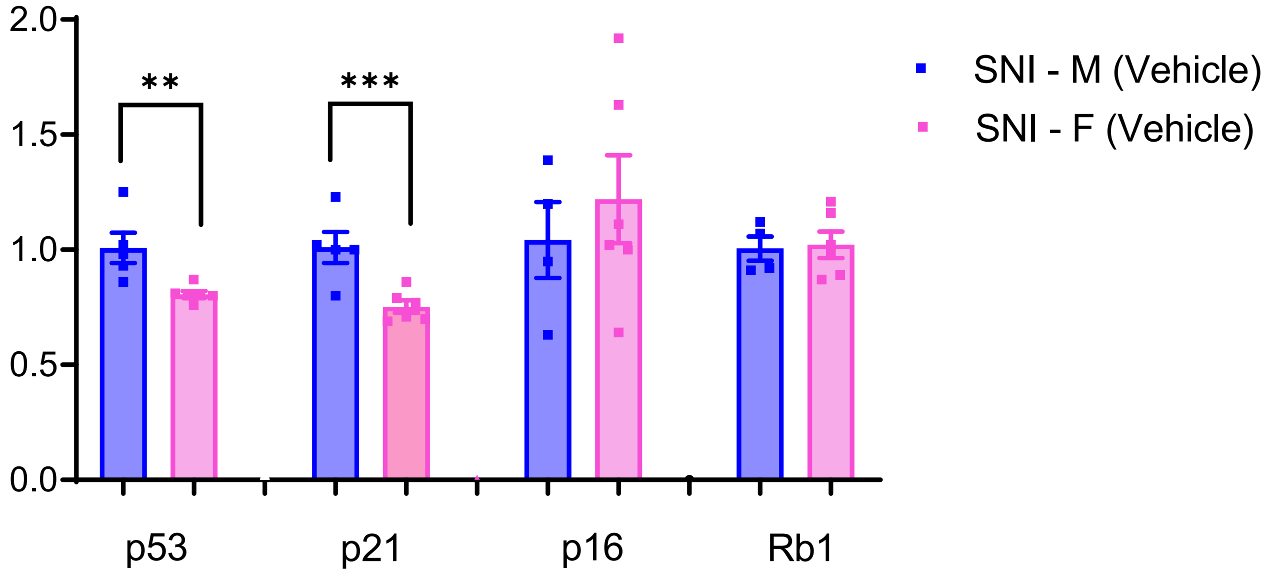

Supplementary Fig. 12. Replication of male- and p53/p21-specific upreglation of senescence-related pathways in SNI-treated mice. Shown are data from vehicle-treated groups shown in Fig. 6c. qRT-PCR was performed on spinal cord tissue obtained from mice 9 months post-SNI and 22 days post-drug (see Fig. 6a). Bars represent mean ±SEM relative expression compared to the housekeeping gene *Gapdh*, and normalized to males (note that in Fig. 5c, drug groups are normalized to same-sex vehicle). The sex difference in p53 (t_9 = 3.2, p=0.01) and p21 (t_8 = 3.8, p=0.005) expression is a direct replication of the data shown in Fig. 4a, as is the lack of sex differences in p16 (t_8 = 0.6, p=0.53) and Rb1 (t_8 = 0.2, p=0.84) expression. **p=0.01, ***p-0.005 as indicated.
